# Lack of Ecological and Life History Context Can Create the Illusion of Social Interactions in *Dictyostelium discoideum*

**DOI:** 10.1371/journal.pcbi.1005246

**Published:** 2016-12-15

**Authors:** Ricardo Martínez-García, Corina E. Tarnita

**Affiliations:** Department of Ecology and Evolutionary Biology, Princeton University, Princeton NJ, United States of America; University of California Irvine, UNITED STATES

## Abstract

Studies of social microbes often focus on one fitness component (reproductive success within the social complex), with little information about or attention to other stages of the life cycle or the ecological context. This can lead to paradoxical results. The life cycle of the social amoeba *Dictyostelium discoideum* includes a multicellular stage in which not necessarily clonal amoebae aggregate upon starvation to form a possibly chimeric (genetically heterogeneous) fruiting body made of dead stalk cells and spores. The lab-measured reproductive skew in the spores of chimeras indicates strong social antagonism that should result in low genotypic diversity, which is inconsistent with observations from nature. Two studies have suggested that this inconsistency stems from the one-dimensional assessment of fitness (spore production) and that the solution lies in tradeoffs between multiple life-history traits, e.g.: spore size versus viability; and spore-formation (via aggregation) versus staying vegetative (as non-aggregated cells). We develop an ecologically-grounded, socially-neutral model (i.e. no social interactions between genotypes) for the life cycle of social amoebae in which we theoretically explore multiple non-social life-history traits, tradeoffs and tradeoff-implementing mechanisms. We find that spore production comes at the expense of time to complete aggregation, and, depending on the experimental setup, spore size and viability. Furthermore, experimental results regarding apparent social interactions within chimeric mixes can be qualitatively recapitulated under this neutral hypothesis, without needing to invoke social interactions. This allows for simple potential resolutions to the previously paradoxical results. We conclude that the complexities of life histories, including social behavior and multicellularity, can only be understood in the appropriate multidimensional ecological context, when considering all stages of the life cycle.

## Author Summary

Fitness in social microbes is often measured in terms of reproductive success in the social stage, with little regard to other stages of the life cycle (e.g. solitary) or to the ecological context. This approach can lead to seemingly paradoxical results that point to complex social interactions (e.g., social cheating) among individuals in the population. However, recent experimental studies in *Dictyostelium discoideum*, one of the most studied social microbes, have highlighted various tradeoffs among previously ignored non-social traits that should affect fitness. We develop an ecologically-motivated socially-neutral model for the life cycle of *D*. *discoideum* that combines these proposed traits and tradeoffs and proposes new ones to determine whether existing observations can be explained without the need to invoke social interactions. We confirm this expectation and conclude that the complexities of social behavior can only be understood in the appropriate ecological context, when considering a complete description of the life cycle.

## Introduction

The cellular slime mold *Dictyostelium discoideum* is one of the most studied examples of cooperation and altruism in microbes. Upon starvation, solitary amoebae aggregate with neighbors to form a multicellular fruiting body made of stalk and spores. The spores are resistant and will germinate upon encountering favorable conditions while the stalk cells die during stalk development [1–4]. In the process of aggregation these amoebae do not exclude non-kin; consequently, chimeras (multicellular fruiting bodies consisting of at least two genotypes) have been observed both in the lab and in nature [5–8]. These chimeras are functional: the multiple genotypes participate both in stalk formation and in spore production (although not necessarily in equal measures, a phenomenon known as reproductive skew [6]). Studies to date have found significant reproductive skew in *D*. *discoideum* chimeras [9,10] and in a variety of other cellular slime molds [11]. This was interpreted as a sign of social conflict and strains that were over-represented in the spores were seen as socially dominant [9,10]. Furthermore, the apparent dominance of some strains over all others (e.g., in *D*. *discoideum*, linear dominance hierarchies were recorded) raised into question the persistence of the latter in the wild. Thus, the experimentally uncovered dominance relationships seemed to point towards a decrease in species-wide genetic diversity that was inconsistent with the immense diversity and coexistence observed among strains in nature [5,8,9,11].

Recent studies [12,13] have suggested that both the impression of social conflict and the inconsistency between experimental predictions and natural observations arise due to the one-dimensional assessment of *D*. *discoideum* fitness, which is equated to spore contribution, when in fact life-history tradeoffs between non-social traits can lead to multiple fitness components. The first proposed tradeoff concerns the response to starvation and lies in the decision between staying vegetative and becoming a spore [12]. In *D*. *discoideum* not all cells aggregate to become multicellular; experiments have shown that these cells are viable [12] and theoretical approaches suggested that they could be part of a bet-hedging strategy in uncertain environments [12,14]: non-aggregators (also called loner cells in [12]) have a high chance of death if the starvation period is long, but, if food does return to the environment, they have a head start against spores that need time to germinate. Given this tradeoff, genotypes that are overrepresented in spores might not be social cheats but rather they could simply be those that have been selected to leave fewer non-aggregators behind [12]. While this tradeoff does not yield coexistence in a well-mixed setting, it can do so in the presence of temporal heterogeneity (e.g., seasonality [15]), or of spatial heterogeneity and spore dispersal [12]. Subsequently, a second tradeoff was determined empirically, between spore number and viability: genotypes that were overrepresented in spores also made smaller and less viable spores when grown clonally, suggesting that the previously-determined social dominance might be only apparent [13]. This tradeoff was hypothesized to allow coexistence of multiple strains [13].

While [12–14] convincingly argue that *D*. *discoideum* fitness has multiple components that, when ignored, can create the illusion of social interactions, there is no immediate link between the two different tradeoffs they propose, nor is there a theoretical framework in which to assess (a) how many such tradeoffs are likely to occur and what are the relationships between them, (b) by what mechanisms they play out and (c) whether they are sufficient to resolve the inconsistency between the lab-based dominance hierarchies and the experimental observations. To this end, we set out to provide such an eco-evolutionary framework in which we study selection on non-social life-history traits in *D*. *discoideum*, explore the possible tradeoffs that can arise, and make testable predictions for future empirical work. While the framework is not all-encompassing, it provides a theoretical starting point on which additional ecological and life-history knowledge can be built. We recapitulate existing experimental results without needing to invoke social interactions, we make testable predictions, and we propose improved experimental designs and measures of *D*. *discoideum* chimeric interactions that capture the variety of tradeoffs.

More broadly, these findings affect our understanding of *D*. *discoideum* social behavior and multicellularity and they resonate with recent studies emphasizing the challenges associated with assessing cooperation and free-riding in microbes in the absence of the ecological context under which the trait deemed cooperative has evolved and is maintained [12–14,16–20]. We showcase some of the complex outcomes (e.g. bet-hedging, coexistence) that ecological context and selection on non-social traits can produce in the absence of social interactions. Many of these outcomes have been theoretically shown before when studied independently (i.e. focus on one trait or one environmental characteristic, e.g. see [21–23]) but here we identify powerful synergistic interactions that cannot be predicted from one-dimensional analyses. Finally, these results also contribute towards clarifying the misinterpretations that can arise from sociobiological investigations into microbe behavior that are not grounded in an ecological understanding.

## Results

### Eco-evolutionary framework

Starting from experimental observations, we build a model of cellular slime molds incorporating hypothesized life-history traits and tradeoffs and their functional consequences [24]. The model we employ is a generalization of [12] to study more broadly life-history tradeoffs in *D*. *discoideum* in response to environmental stressors. Since we are interested in nonsocial traits, we study a well-mixed population in which we assume that there are no social interactions between genotypes and in which we do not model the spatial aggregation process. This model therefore ignores interactions between genotypes that may arise during the developmental process. Although *D*. *discoideum* and other cellular slime molds are likely to be found in spatially structured environments where movement in the vegetative state is limited, a first well-mixed approach is necessary to tease apart the effects of spatial structure from effects arising from simple life history tradeoffs in a well-mixed setting. In this socially neutral, well-mixed context, aggregation occurs randomly (with anyone in the population) and within chimeric aggregates there are no interactions.

#### Environment

**Fig 1:**
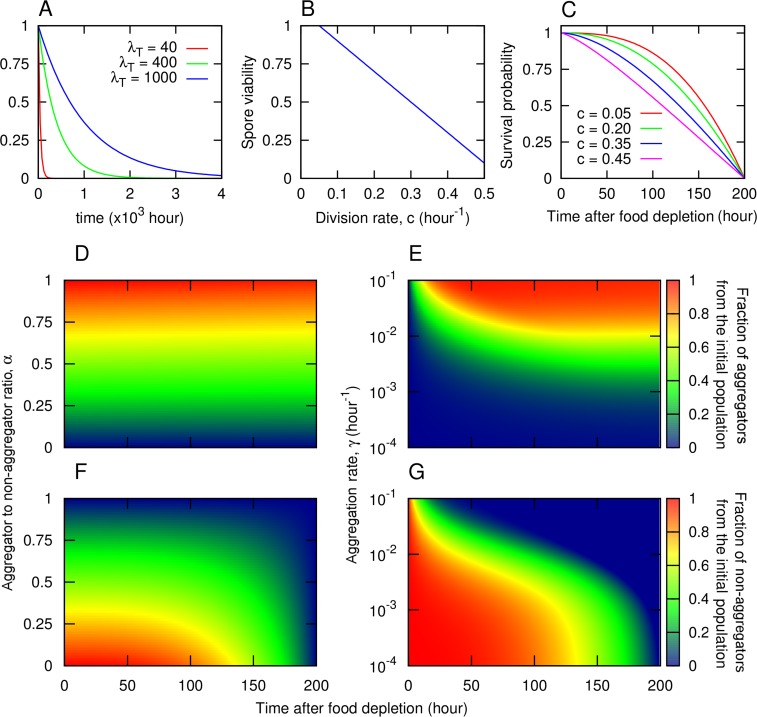
Framework assumptions. A) After food consumption, the time until the arrival of a new pulse of nutrients is a stochastic variable that follows an exponential distribution of mean value *λ*_T_ (not normalized for clarity). B) Spore viability decreases linearly with division rate, which is assumed to correlate negatively with cell size. C) Survivorship curves for starving non-aggregated cells with different division rates, *c*. The curves show the probability of being alive at a time *t* after starvation, which correlates negatively with the division rate. For D)–G) we used the survival curve corresponding to *c* = 0.15. D, F) Discrete aggregation mechanism. Genotypes are determined by the fraction of the population, *α*, that aggregates. The rest, a fraction 1 – *α*, remains solitary. The population partitioning takes place instantaneously after food consumption. E, G) Continuous aggregation mechanism. Genotypes are determined by the rate at which cells aggregate, *γ*. The fraction of the population that aggregates depends both on the aggregation rate, γ, and on the length of the starvation period. D, E) Fraction of the population at the beginning of the starvation period that has turned into aggregators at time *t* after starvation. F, G) Fraction of the initial population that remains as solitary cells at time *t* after starvation. Aggregated cells also die at a very small rate, δ, but this effect is imperceptible at short times scales. Other parameters are specified in S1 Table.

Here we focus on starvation and we study both deterministic environments–in which food recovery is certain and the starvation times (time between onset of starvation and the next food pulse) are always of the same length, *T*, and stochastic environments–in which food recovery is variable and uncertain and the starvation times are drawn from an exponential distribution with average *λ*_*T*_ (Fig 1A). We assume that all cells in a given environment compete for the available resources.

#### Life-history traits

Based on experimental observations, three traits seem to play an important role: the commitment to aggregating versus not aggregating (staying solitary) in response to starvation [12,14]; the spore size; and the spore viability [13]. The first trait, the response to starvation, encapsulates a genotype’s decision to commit resources to sporulation (which occurs as a result of aggregation) or to remaining vegetative, and captures the tradeoff between resistant spores that are able to survive in harsh conditions but are slow to germinate when the conditions improve, and non-resistant solitary cells (non-aggregators) that eventually die in harsh conditions but which are able to immediately start eating and dividing if food returns. The second and third traits–spore size and spore viability–are correlated: spore viability appears to be a direct functional consequence of spore size [13]. This agrees with work in other species where egg or seed size determine germination ability [25–28]. Furthermore, spore size is naturally linked to cell size, the latter being a determinant of cell survival [29]. We hypothesize that cell size, and implicitly spore size, are linked to another trait—the rate at which cells divide, *c*,—via a classical growth-reproduction tradeoff [20]. Given the relationship between size and survival/viability, this further results into a reproduction-survival tradeoff between cells that divide fast and are able to use up common resources but pay the cost of a decreased survival (for non-aggregators) or viability (for spores, [13]), and cells that divide slowly thereby potentially losing out resources to faster dividing strains but at the same time having an increased survival or viability (Fig 1B and 1C).

#### Implementation of the aggregator versus non-aggregator tradeoff

For the aggregators:non-aggregators tradeoff, it has been hypothesized that a stochastic switch is responsible for its implementation [12,14], such that, upon starvation, the population splits instantaneously into fixed fractions of aggregators (some of which become spores while others die during stalk formation) and non-aggregators. In this case the trait under selection is the fraction of individuals that aggregate upon starvation, *α* (Fig 1D and 1F): if *α* = 0, a monoculture of genotype *α* does not undergo aggregation; if *α* = 1, a monoculture only produces aggregators and leaves no non-aggregators behind. Intermediate *α* values represent a mixed strategy where some cells aggregate and others remain solitary. However, there is no experimental proof that a discrete mechanism such as a stochastic switch is responsible for the non-aggregators. Therefore, in addition to the stochastic switch, we also study a second, continuous mechanism, whereby starving solitary cells turn into aggregated cells continuously at rate *γ*, which is the trait under selection. The higher the rate *γ*, the faster the cells aggregate (Fig 1E and 1G). While in the discrete approach only a certain fraction of cells can aggregate and the others remain solitary, vegetative cells, in the continuous approach every cell has the potential to aggregate and become a spore, but some take longer than others. Therefore, for a given genotype (*γ*, *c*), the final number of spores is determined by the length of the starvation period (Fig A(i) in S1 Text). This latter approach also allows the study of the time to complete aggregation (henceforth aggregation time), defined as the time it takes for all the non-aggregators either to die or to aggregate. With this definition, the lower the aggregation rate *γ*, the longer it takes a genotype to complete aggregation (Fig A(ii) in S1 Text). In what follows we will show that the model predictions are robust to the choice of mechanism; therefore, to simplify the presentation, we will focus on the discrete mechanism in the main text and present the continuous mechanism in the S1 Text.

#### Model

The dynamics of the free-living amoebae is given by a well-mixed model of competition for a resource *R* between the different genotypes, each given by a pair (*α*, *c*) or (*γ*, *c*), depending on the mechanism under investigation. The genotypes have abundances X_*,*c*_, where the _*_ is a place holder for either *α* or *γ*, depending on the mechanism under investigation. Thus the dynamics of the populations at this stage are given by,

X˙*,c(t)=cR(t)R12+R(t)X*,c(t)

R˙(t)=−R(t)R12+R(t)∑*,ccX*,c(t) (1)

Resources are consumed according to Michaelis-Menten kinetics with saturation constant *R*_1/2_. When food is abundant, growth rates are related to population doubling times, *T*_*d*_, by *c* = *ln2/ T*_*d*_. We assume that in the growing phase cell death rate is zero. This is a good approximation since most cellular death is caused by starvation. Cells eat and divide as long as resources are available; when resources have been exhausted, cells enter a starvation phase, whose length is determined by the environmental conditions and might be constant (deterministic environments), or sorted from an exponential distribution (stochastic environments). Finally, we use a sum in Eq (1) due to the discretization of the trait space, necessary for computational feasibility; it would be replaced by an integral when considering the continuous trait space.

During the starvation phase, spores and non-aggregators die at different rates. Spores are dormant and die at a constant rate while non-aggregators die according to survival curves that depend on the cell size, which we assume to be inversely correlated with the division rate *c* (Fig 1C). Experimental results have demonstrated that in the absence of food non-aggregators survive by consuming their own organelles and cytoplasmic resources via autophagy [30]. This results in a low mortality rate at short times. Once they consume all the intracellular resources the mortality rate increases and finally all non-aggregators die after a maximum lifetime. We propose that, by analogy with life history tradeoffs in other species [21], strains that reproduce faster have smaller cells, with fewer cytoplasmic resources and consequently have a higher mortality rate at short times. Reproducing faster has therefore an inherent cost in terms of a less mature and more vulnerable offspring. We model this by choosing a family of survival curves (Fig 1C) with different short-time decays for every reproductive rate,

$$S_{c}(t)=\frac{e^{-{\left(\mu t\right)}^{\beta (c)}}-e^{-{\left(\mu T_{sur}\right)}^{\beta (c)}}}{1-e^{-{\left(\mu T_{sur}\right)}^{\beta (c)}}}$$ (2)

where *μ* is the speed at which the death rate of a non-aggregated cell changes with time since starvation and *β*(*c*) is a function of the reproductive rate accounting for the cost of the reproduction speed (*β* > 1 to ensure a slow decay at short times, consistent with [29]). Therefore, *S*_*c*_(*t*) gives the probability of being alive at time *t*. Due to the lack of experimental data for the survival curves, we picked *β* satisfying the assumption that it is a decreasing function of the reproduction rate, reflecting that strains with a higher reproduction rate have to pay a cost in terms of high short-time death rates (smaller values of *β* result in survival curves that decay faster at short times). For the analysis in the main text we use *β*(*c*) = 3.1−4*c* which leads to the survivorship curves in Fig 1C. However, we also performed a sensitivity analysis for the choice of *β* (S3 Fig and S4 Fig), which showed that the shorter the interval in *β* (more similar survivorship curves as shown in S4 Fig) the faster reproduction is favored in a given environment (S4 Fig, bottom). This is the expected result, since having similar survivorship curves while keeping fixed the differences in the reproduction rates, reduces the relative cost of reproducing fast while keeping fixed the benefits.

Population partitioning between aggregators and non-aggregators also takes place during the starvation phase. As described above, we explore two different mechanisms: a discrete mechanism, which we present below, and a continuous mechanism, which we present in S1 Text. In the discrete case (e.g. stochastic switching) aggregators and non-aggregators are determined instantaneously after starvation onset. For a genotype *α* a fraction *α* of the cell population aggregates to eventually form spores and the remaining fraction, 1 − *α*, stays as solitary cells. However, not all aggregated cells become spores–a fraction of them die in the process of stalk formation. Although stalk investment is a crucial component of *D*. *discoideum* life cycle, here we do not study its evolution; this is because stalk investment–a trait considered to be important for dispersal–is not meaningful in the well-mixed scenario under study. Therefore, consistent with experimental results that find a constant 20%:80% stalk:spore ratio across varied experimental settings [1], we multiply the number of aggregated cells by a parameter *s* = 0.8, independent of the genotype, to obtain the number of spores (and thus account for the cells that are lost in the formation of the stalk). During the starvation period only cell death occurs. Spores are dormant and die at a constant, very low rate δ; non-aggregators die according to the survival curves detailed above, such that

$${\dot{X}}_{\alpha ,c}^{sp}(t)=-\delta X_{\alpha ,c}^{sp}(t)\;{\dot{X}}_{\alpha ,c}(t)={\dot{S}}_{c}(t){\tilde{X}}_{\alpha ,c}$$ (3)

where ${\tilde{X}}_{\alpha ,c}$ is the initial population of the strain (*α*, *c*) at the beginning of the starvation phase.

The starvation phase is followed by a new growth phase induced by the arrival of the next food pulse of magnitude *R*_0_ = *10*^*8*^. Then, non-aggregators surviving the starvation phase start reproducing immediately according to Eq (1). The spores, however, start their germination process lasting for a time τ, and only after its completion they are able to start eating and dividing again. Different genotypes will have different germination success, ν, due to their characteristic spore viability, which is dependent on spore size [13]. Consistent with existing work in *D*. *discoideum* [13] and in other species [25–28], we assume that the spore viability is positively correlated with cell size, which, in turn, we hypothesized to be inversely correlated with the division rate, *c*. Thus, for simplicity, we assume the spore viability to be a decreasing linear function of the reproduction rate *c* (Fig 1B):

ν(c)=1.1−2c (4)

Strains with the fastest reproduction have the lowest spore viability *v* = 0.2; at the other extreme, strains with the slowest reproduction have the highest spore viability, *v* = 1 (Fig 1B).

Our results are structured in two categories (Table 1). First we run (a) evolutionary simulations to determine the equilibrium population composition in a given environment (here growth—starvation cycles continue indefinitely and the lengths of the starvation periods are either fixed in the deterministic case or drawn from an exponential distribution in the stochastic case). We find that each environment selects for only one winning genotype, suggesting that the presence of multiple traits and tradeoffs is not sufficient to ensure coexistence of multiple genotypes. Second, since they are the only surviving genotypes in our simulations, we take the winning genotypes from each environment and run simulations that replicate experimental setups (either one starvation event or one growth-starvation event) to determine whether we can recapitulate existing experimental results. Here we study both (b) properties of individual strains and (c) properties of strains within chimeric mixes.

**Table 1:** Summary of the model and results.

Exp. observations	I. Hypothesized trait involved	II. Hypothesized tradeoff	III. Functional consequence	Predictions of the socially-neutral model incorporating I-III in stochastic environments		Exp. support
Non-agg exist and are viable [12,14]	Aggregator to non-agg. ratio *α*	Non-agg. versus spore production	Non-agg.: less resistant to starvation; reproduce readily if food returns. [12,14]	(a) Evolution	One winning genotype selected per environment	Untested
					Longer starvation times select for more spores	Untested
					SE and FE: longer starvation times select for faster reproducers; IE: the opposite holds.	Untested
			Spores: resist starvation; delayed reproduction if food returns. [1]	(b) Correlations b/w non-social traits	*After one growth-starvation*: - spore number ↗ with *c* for SE; ↘ for IE; **=** for FE. - opposite holds for non-agg	Untested
Genotypes with more spores, have smaller, less viable spores [13]	Division rate *c*	Growth versus reproduction versus survival	Cell/spore size anti-correlates with *c*	(c) Correlations with chimeric success (CS)	Linear hierarchy of genotypes based on CS (regardless of measure)	Agrees w/ [9,10]
	Cell/spore size		Cell/spore size correlates with cell survival/ spore viability. [13,29,30]		*After one growth-starvation* -:CS ↗ *α* for SE and FE, ↘ *α* for IE. CS ↗ *c*. - CS ↘ spore size.	Untested
	Cell survival/ spore viability				*After starvation only*: - CS ↗ *α*. CS ↗ *c* for FE, ↘ *c* for IE, and **=** for SE. - CS ↘ spore size for FE, ↗ for IE, and **=** for SE.	Broadly agrees w/ [13]

Starting from experimental observations, we build a socially-neutral model (i.e. no social interactions between genotypes) of cellular slime molds incorporating hypothesized life-history traits and tradeoffs and their functional consequences [24]. The environment is characterized by its stochastic distribution of starvation times (i.e. the time between food pulses); SE = slow-recovery environments; IE = intermediate-recovery environments; FE = fast-recovery environments. For precise definitions and details see the text. ↗ denotes positive correlation; ↘ denotes negative correlation; = denotes constant. Evolutionary simulations are run over a very large number of growth-starvation cycles. Correlations between traits and correlations between chimeric success and various traits are determined after one growth-starvation cycle, for which depending on the initial conditions (cell-to-food density), the growth period can range from very long to inexistent.

### Evolutionary simulations in deterministic environments

**Fig 2:**
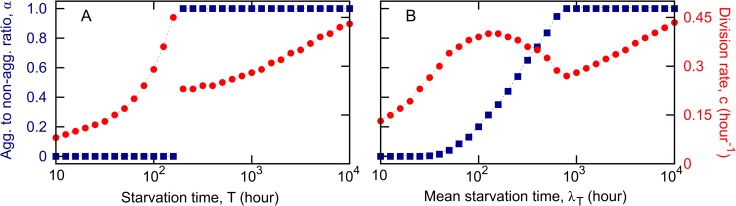
Winning genotypes in deterministic and stochastic environments. In both panels blue squares represent the aggregator to non-aggregator ratio and red circles represent the division rate; dashed lines are interpolations. Simulations are initialized with a number of genotypes that compete for a pulse of resources through several growth-starvation cycles. Long realizations show that only one strain (which we call the winner) is able to survive in the stationary state in a given environment. For computational feasibility, the winner is determined as the most abundant genotype at *t =* 10^8^, when a few genotypes still survive. A) Deterministic environments, simulations are initialized with 4141 genotypes (101 values of *α* and 41 values of *c*). B) Stochastic environments. Results averaged over 20 independent simulation runs initialized with 44772 genotypes (1092 values of *α* and 41 values of *c*). Parameters as in S1 Table.

In a given environment, only one genotype emerges as a winner if given long enough time. For computational optimization, the winning genotype was determined as the most abundant after *t =* 10^8^ hours in the simulations, when a few genotypes are still present in the system. Longer realizations, however, show that the rest of the genotypes are outcompeted and only one strategy survives, consistent with [12,14]. Also consistent with previous results [12,14], deterministic environments always select for pure strategies: environments where food recovers faster select for all non-aggregators (*α* = 0) and environments where food recovers slower select for all spores (*α* = 1) (Fig 2A). The switch between these two regions (all-non-aggregators versus all-spores) takes place at starvation times close to the maximum lifespan of a non-aggregator in the absence of food. In both regions, the lower the starvation time, the lower the growth rate *c*. In the first region (selection for all-non-aggregators), due to the very short starvation periods, the whole population increases over time so that each new growth period starts with a higher initial cell density. Since we assume that the food pulse is always the same size, then an increasing population finishes the food faster and thus reduces the length of the growing periods. The shorter the growing periods, the less advantage for the fast reproducing strains. Hence, for very short starvation periods, fast reproducing strains have an initial growth advantage but, over many growth-starvation cycles, they are outcompeted by slow reproducing ones as growth benefits diminish and are outweighed by the higher survival costs incurred during the starvation phase (S1 Fig, panels A, B). As the length of the starvation periods increases, faster reproducing strains start to win (Fig 2A, S1 Fig, panels A, C). In the second region (selection for all-spores), we find the same increasing trend in *c* as the length of the starvation period increases (Fig 2A). This is due to the fact that few spores die during short starvation times and therefore, the initial population sizes responding to a new food pulse are large and able to consume the food quickly. This results in short growth periods; consequently, strains that reproduce faster do not get enough divisions during a growth cycle to overcome the cost of having a lower spore viability (S1 Fig, panels A, D).

### Evolutionary simulations in stochastic environments

Stochastic environments also select for only one surviving genotype, which was identified in simulations as in the deterministic case above. Consistent with previous results [12,14] as well as extensive theoretical work on bet-hedging (e.g. [22,31]), this winning strategy is pure at the extremes–all-non-aggregators for very fast and all-spores for very slow environments–but mixed otherwise (Fig 2B). This holds regardless of whether we use a discrete or a continuous spore-forming mechanism. In this case the growth rates follow similar trends to the deterministic case for fast and slow environments but follow the opposite trend for intermediate environments (Fig 2B). Henceforth, we will call intermediate environments precisely those for which the reproduction rate of the winning genotype decreases with increasing length of starvation; environments to the left of this region will be called fast and environments to the right of this region will be called slow. The intuition behind the increasing trend in fast and slow environments is the same as for the deterministic case (S2 Fig, panels A, B, D). For intermediate environments, the decreasing trend in division rate of the winning genotype occurs when the starvation periods are long enough to be costly for the survival of non-aggregators but they are still short so that spores incur little mortality leading to short growth periods during which fast reproducing cells do not derive enough benefit to offset the lower viability of their spores (S2 Fig, panel C). For realistic values of the spore death rate (not too large), these qualitative trends are robust with respect to parameter choice both in the stochastic and in the deterministic case (S3 Fig and S4 Fig). The results are independent of the initial conditions (S5 Fig, panel A) but they are affected by the saturation behavior assumed for the Michaelis-Menten dynamics (S5 Fig, panel B).

In what follows we will focus on stochastic environments since they are more likely to capture the realities of microbial lives [32]. When we refer to fast-environment, intermediate-environment or slow-environment genotypes, we mean the winning genotypes from fast, intermediate, respectively slow environments.

### Correlations between non-social life-history traits

**Fig 3:**
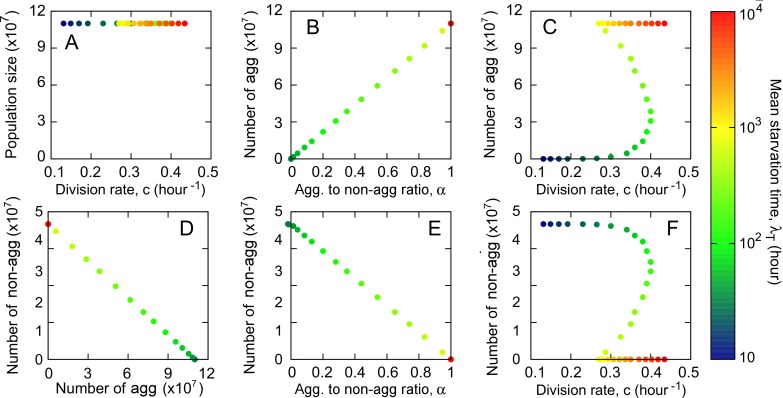
Correlations between non-social traits. A clonal growth period for each one of the 31 winning genotypes obtained in Fig 2B is integrated to evaluate correlations between the non-social traits included in the model at the onset of starvation. A) The total population is constant for all genotypes. Number of aggregators versus B) the aggregator to non-aggregator ratio, *α*; C) division rate, *c*. Number of non-aggregators versus D) the number of aggregators; E) the aggregator to non-aggregator ratio; F) the division rate.

To determine the correlations that emerge between reproduction, survival and viability as a function of the traits and tradeoffs included in our model, we used the winning genotypes from each stochastic environment (Fig 2B) and we allowed initial clonal populations of identical size to complete one growth phase on identical resources and undergo the sporulation process triggered by starvation upon resource depletion. Thus, unlike in our evolutionary setup above where competing genotypes underwent many successive growth-starvation cycles, for the purposes of exploring correlations between non-social traits we replicated laboratory setups of one growth period followed by the subsequent starvation to allow for comparison with existing data. Since the population partitioning between aggregators and non-aggregators followed by the spore:stalk cell differentiation takes place in our model instantaneously upon resource depletion, we do not need to consider the duration of the starvation phase; all quantities of interest are evaluated at the onset of starvation. We determined the total population size, number of aggregators and number of non-aggregators for each of these genotypes and related them to each other, to the division time *c*, and to the investment in aggregators versus non-aggregators, *α* (Fig 3).

At the end of a growth period started from identical and monoclonal initial conditions the population size is constant with respect to *c* (Fig 3A). This follows from the assumption that resources are used proportional to the division rate and that there is no (or negligible) cell mortality during the growth phase: i.e., strains that divide faster (higher *c*) use more resources per division and therefore reach their carrying capacity sooner, while strains that divide slower (lower *c*) use fewer resources per division and reach their carrying capacity later. However, since all strains start with identical numbers on identical resources, when the resources are exhausted they all reach the same carrying capacity. Thus, when strains grow clonally for only one growth period, higher division rate does not confer a benefit in terms of increased number of cells; however, it continues to incur a cost during starvation in terms of cell survival. This is an essential difference from the evolutionary setup where mixes of several strains compete for resources during many growth-starvation cycles.

As a consequence of the hypothesized tradeoff between aggregator and non-aggregator production, the number of aggregators is equal to *αP* and the number of non-aggregators is equal to (1-*α*)*P*, where *P* is the population size at the end of the growth phase, which, as discussed above, is independent of *α* and constant with respect to *c*. Thus, the number of aggregators always increases with *α* (Fig 3B) and it has the same behavior with respect to *c* as does *α*: i.e. the production of aggregators increases with the division rate *c* for slow-environment genotypes, decreases with *c* for intermediate-environment ones and is constant with respect to *c* for fast-environment genotypes (Fig 3C). Aggregators and non-aggregators are anticorrelated, so that a high investment in aggregators comes at the cost of a low investment in non-aggregators (Fig 3D). Non-aggregators always decrease with *α* (Fig 3E) and with respect to *c* they have the inverse behavior of *α*: i.e. non-aggregator production decreases with *c* for fast-environment genotypes and increases for intermediate ones (Fig 3F). Slow-environment genotypes do not produce non-aggregators.

It is important to note that these results are sensitive to the experimental/simulation conditions. All results above hold if monocultures are allowed to undergo a full growth-starvation cycle. If, as has been the case in experimental studies [13], monocultures are grown exponentially in abundant resources and then washed and abruptly starved, we find an overall positive correlation between number of spores and division rate (S6 Fig). This yields a negative correlation between number of spores and cell and spore size that agrees with experimental findings reported in [13].

### Chimeric success

In this neutral context where individuals do not interact with each other except indirectly via their competition for resources (possible inter-strain interactions during the aggregation and the development of the fruiting body are neglected), chimeric success is only apparent and it is simply a measure of which genotype makes more spores in a mix. So far chimeric success has been measured at the end of one starvation event (i.e. no mixed growth period) [10,11,13]; however, genotypes that starve together in nature must have shared at least one growth period (i.e. the one prior to the starvation event). Therefore, in the context of our new understanding of the interplay between life history traits we propose a new measure of chimeric success that emphasizes the importance of both growth and starvation. Since the natural history of slime molds is insufficient to infer how many such growth-starvation cycles two genotypes are likely to share, we restrict our measure of chimeric success to only one growth-starvation cycle. Thus, chimeric success is not a measure of fitness, but rather a measure of spore production in a mixed context. Finally, although chimeras made of several genotypes occur in nature occasionally, experimental work has exclusively focused on pairwise mixes and therefore for ease of comparison we will limit our analysis to this case.

The difference between our proposed measure of chimeric success and the existing one consists in the setup: for the existing measure, which we will denote CS_S_, one starts with a 50:50 mix of two starving genotypes and allows them to aggregate and form spores; for the new measure, which we will denote ${\text{CS}}_{\text{GS}}^{X_{0}}$, one starts an initial population of size *X*_0_ composed of a 50:50 mix of two genotypes on an amount of food, *R*_0_, and allows the cells first to grow, then starve naturally, and subsequently aggregate and form spores. In both setups, in keeping with previous work [9,10,13], the chimeric success of a genotype in a pairwise mix is given by its fraction of the total spores; the overall chimeric success of a genotype is the average over all such pairwise mixes. For mathematical definitions and details see Methods. Although the two setups are different, the measure we propose reduces to the existing one in the limit of high cell relative to resource density, when food is insufficient to support growth and therefore cells starve instantaneously.

The chimeric success is measured upon completion of spore formation. Thus, for the discrete spore formation mechanism chimeric success is measured at the onset of starvation, when the aggregate and the spores are formed instantaneously; for the continuous spore formation mechanism, the number of spores has to be measured after a standardized starvation time, at which all genotypes will have completed their aggregation. We therefore measure the quantities of interest at time *T*_*sur*_ = 200 hours of starvation, which is the maximum amount of time that a starving solitary cell can live. A more extensive discussion of the continuous mechanism is shown in S1 Text.

**Fig 4:**
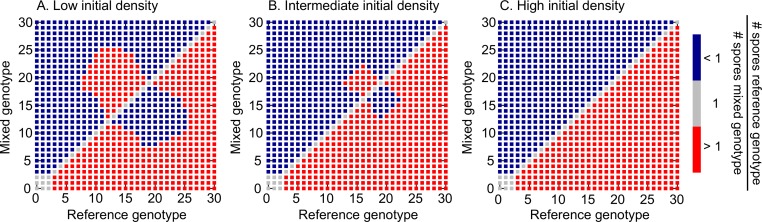
Relative chimeric success in pairwise mixes. The winning genotypes from Fig 2B are mixed in pairs and their relative number of spores measured after a single growth-starvation cycle. Genotypes are ordered according to the environment where they evolved in, with 0 corresponding to the fastest-recovery environment (i.e. *λ*_*T*_ = 10 hours) and 30 corresponding to the slowest-recovery one (i.e. *λ*_*T*_ = 10^4^ hours). To define chimeric success, we refer to one of the genotypes in the mix as reference genotype (x-axis) and to the other as mixed (genotype). Mixes in which the reference genotype produces more spores than its mixed partner are represented by red squares, whereas blue squares represent mixes in which the mixed genotype produces more spores. Mixes in which both genotypes produce the same amount of spores are represented by gray squares. A ranking of the genotypes according to their chimeric success is determined using the number of pair mixes in which a given genotype produces more spores than its partners; this depends on the initial amoebae relative to resource density. A) Low initial cell:resource density: 10^3^ cells and *R*_0_ = 10^8^ resources, B) Intermediate initial cell: resource density: 10^7^ cells and *R*_0_ = 10^8^ resources, C) High initial cell:resource density: 10^10^ cells and *R*_0_ = 10^8^ resources.

**Fig 5:**
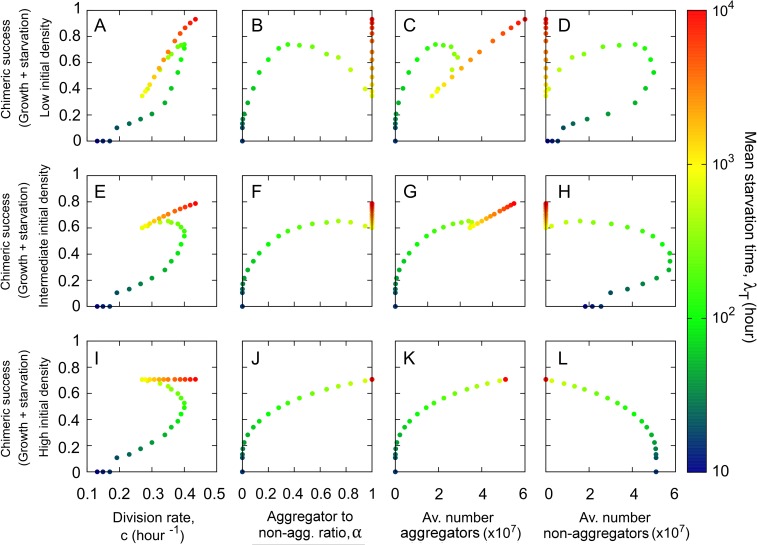
Correlations between overall chimeric success and non-social life history traits. Each one of the 31 winning genotypes obtained in Fig 2B is mixed in pairs with the rest of the genotypes and the fraction of spores is counted following a growth phase and the subsequent starvation onset. The chimeric success is obtained as the mean value of this fraction of spores averaged over all the possible pair mixes as defined in Eq (5) of the main text. From left to right: chimeric success versus division rate (A, E, I), aggregator to non-aggregator ratio (B, F, J), average number of aggregators (C, G, K) and average number of non-aggregators (D, H, L). From top to bottom: low cell:resource initial density (10^3^ cells and R_0_ = 10^8^), intermediate cell:resource initial density (10^7^ cells and R_0_ = 10^8^) and high cell:resource initial density (10^10^ cells and R_0_ = 10^8^).

As already outlined above, since each environment only selects for one genotype, we assume that, at equilibrium, genotypes that can end up in mixes are these winning genotypes, perhaps as a result of dispersal between different environments. Therefore, for the pairwise comparisons we used the winning genotypes from each environment obtained in Fig 2B and started with 50:50 mixes of low (Fig 4A and Fig 5 top row), intermediate (Fig 4B and Fig 5 middle row) and high (Fig 4C and Fig 5 bottom row) initial cell densities relative to resource magnitude. As before, in what follows, when we refer to fast-environment, intermediate-environment or slow-environment genotypes, we mean the winning genotypes from these respective environments. We want to investigate two aspects. First, we want to explore relative chimeric success between pairs of genotypes (Fig 4). Second, we want to determine how overall (average) chimeric success correlates with non-social life history traits: division time *c* and implicitly cell survival and spore viability, spore production, aggregators to non-aggregators ratio and number of non-aggregators (Fig 5). Because in mixes that undergo a growth period the production of spores and non-aggregators depends on the mixing partner, we compare the chimeric success of a genotype not to its production of spores and non-aggregators in monoculture, as determined in the Correlations between non-social life-history traits section, but to its average production of aggregators and non-aggregators, where the average is taken over all pairwise mixes.

As explained above for clonal growth, in one growth-starvation cycle the spore-forming mechanism does not affect the growth period. Unlike in the case of monocultures, however, where the population size is constant with respect to *c* at the end of growth, in mixes, a higher division rate *c* gives the benefit of faster food consumption and increased population size. Although higher *c* still incurs the cost of higher mortality during starvation, if the growth period is sufficiently long, its potential benefits can outweigh the costs. Since chimeric success is determined upon starvation-induced fruiting body formation following one growth period, what matters is the length of the growth period. This depends on the initial cell density relative to the amount of available food such that the lower the initial cell density relative to the food pulse, the longer the growth period.

Both in the pairwise and in the average scenarios we find that, at low cell-to-resource initial densities, chimeric success is mainly determined by growth and therefore its behavior is dominated by the division rate rather than the aggregator to non-aggregator ratio. As the initial cell density relative to resource magnitude is increased, the two forces–division rate and aggregator to non-aggregator ratio–start to equilibrate and eventually, at high cell-to-resource density where there is only residual growth, the starvation period dominates and the chimeric success is mostly determined by the aggregator to non-aggregator ratio.

For pairwise comparisons, regardless of the initial density we find that our strains are organized in a linear hierarchy of chimeric dominance, consistent with lab results for *D*. *discoideum* [9,10] (Fig 4). However, the ranking of the strains in the hierarchy, given by the number of pairs (red squares in Fig 4) in which the focal strain produces more spores than its partner, strongly depends on initial cell-to-resource densities. For high initial cell densities, the hierarchy ordering is dominated by the spore investment and we find that the higher the *α*, the higher the chimeric success of the genotype (Fig 4C). As the initial cell density decreases and the division rate starts to play a stronger role, intermediate-environment genotypes for which spore investment is anticorrelated with the division rate separate into two symmetric groups that interchange their places in the hierarchy (Fig 4A and 4B): the group with the higher *α* and lower *c* gets displaced by the group with the lower *α* and higher *c*. The slow-environment genotypes continue to top the hierarchy and the fast-environment genotypes continue to be at the bottom of the hierarchy.

Moving on to the overall (average) chimeric success, we find that at low initial densities it is positively correlated with the division rate (Fig 5A); it is positively correlated with the average number of spores for fast- and slow-environment genotypes but it is negatively correlated with it for intermediate-environment genotypes for which the negative effect of decreasing *c* is stronger than the positive effect of increasing *α* (Fig 5B and 5C). Finally, the behavior of the chimeric success with respect to the number of non-aggregators is the opposite of the behavior with respect to spores (Fig 5D).

As the initial cell density relative to resource magnitude is increased, the division rate and aggregator to non-aggregator ratio start to play more equal roles (Fig 5E–5H) and eventually, at high cell density, the latter dominates. Thus, at the high density extreme where growth is only residual and starvation dominates, chimeric success correlates positively with the aggregator to non-aggregator ratio (Fig 5J), and with the number of spores (Fig 5K), and it correlates negatively with the average number of non-aggregators (Fig 5L). Chimeric success still correlates positively with division rate for fast-environment genotypes but it correlates negatively with division rate for intermediate-environment genotypes where now the effect of *α* dominates the effect of *c* (Fig 5I). For slow-environment genotypes where the spore investment *α*, is fixed (maximal), the correlation with division rate is slightly positive, since a higher division rate implies a higher production of spores.

## Discussion

We built a theoretical framework, focused on *D*. *discoideum* but easily generalizable to other sporulating microbes, to explore the effects of selection on non-social life history traits in variable environments. Within this framework, we were able to qualitatively recapitulate existing results attributed to apparent social behavior in *D*. *discoideum* in a model that assumes no social interactions and does not consider any other interaction that may occur during the development of the fruiting body. This highlights the importance of an extended understanding of ecology and life history. In the absence of ecological knowledge–which environment each genotype evolved in, and in the absence of life history knowledge–which life history traits selection acts on and what relationships exist between them, one can incorrectly interpret differences in spore investment as chimeric success and attribute the latter to complex interactions (e.g. social cheating). In the neutral context, previously paradoxical findings do not even arise.

Specifically, we assume that two genotypic traits are under selection–division rate and aggregator to non-aggregator ratio (the latter yielding an immediate anticorrelation between spore number and non-aggregator number). We find a negative correlation between spore number and aggregation time (see S1 Text for details). Furthermore, depending on the simulation setup (encapsulating different experimental setups), additional correlations can be identified between spore production and cell and spore size, survival and viability, such that spore production generally comes at a cost of spore viability, consistent with [13].

In this multi-trait context, measures that attempted to quantify success upon starvation–such as spore number and chimeric success, were revealed to be ill-defined, as we showed starvation and growth to be inextricably linked. We therefore proposed a new and more general measure of chimeric success that accounts for both growth and starvation and found that genotypes are organized in a linear hierarchy based on their pairwise chimeric success, consistent with experimental results [9,10]. Furthermore, the overall chimeric success of a genotype, measured as an average over pairwise comparisons, generally increases with the division rate (i.e. decreases with cell/spore size) consistent with experimental results [13], it increases with the average number of spores produced and with the aggregator to non-aggregator ratio, and it decreases with aggregation time (see S1 Text for details). Finally, we found that the relationship between chimeric success and investment in non-aggregators depends on the experimental conditions. Our results are robust with respect to the choice of mechanism underlying the non-social traits (e.g. stochastic switching versus phenotypic variation).

Despite the multiple life history traits and associated tradeoffs, however, we found that each environment, whether deterministic or stochastic, selects for only one winning genotype. Coexistence can be achieved in the current model if we incorporate spatial heterogeneity and weak-to-moderate dispersal between different environments (consistent with [12]), or temporal heterogeneity [15]. In the absence of any spatial or temporal heterogeneity, multiple genotype coexistence is possible only when strains balance their tradeoffs so that they have identical fitness, as suggested in [13]. Such balancing however reduces the scenarios where coexistence may occur and yields them ungeneric. This is in agreement with classic works in community ecology that show that in the absence of frequency-dependent mechanisms tradeoffs alone do not generally result in coexistence [33].

Several future directions arise. First, our results constitute general testable predictions (Table 1) and emphasize the importance of standardized measures and experimental protocols, ideally chosen to most closely approximate natural conditions. Future work needs to build on existing work in life-history theory that highlights that the reproductive output of just one life-stage is not necessarily an appropriate fitness measure [34–36], to propose a new measure of *D*. *discoideum* fitness, that accounts for the way natural selection acts on the life histories of individuals and their ancestors, in variable environments. Recent work has proposed a very elegant framework in bacteria that could be adapted for other microbes [37]. Second, although we were able to qualitatively capture existing results in a well-mixed, neutral framework, this dismisses neither the importance of spatial structure, which is likely to influence the dynamics at least quantitatively, nor the possibility of social interactions in general. While the linear hierarchies in *D*. *discoideum* are well aligned with the neutral hypothesis, other slime mold species have exhibited short dominance loops [11], possibly indicative of small subsets of interacting genotypes. Even in *D*. *discoideum*, there exists at least one extreme lab mutant that has been shown to interact in chimeras, a social parasite that is unable to form its own stalk but uses other genotypes’ stalks to support its spores [38]. Although such a mutant has not been found in nature, it has been shown to be very destructive in lab experiments [38] and therefore its effects are worth re-investigating using this new multi-trait understanding of fitness. One hypothesis is that the non-aggregators can act as buffers against the destructive effects of a parasite that can only exploit the social aspects of the behavior. This is consistent with existing results from the theory of cooperation and sociality showing that individuals that do not participate in the social dynamics are of primary importance for the evolution of social behavior [39,40]. Third, the life cycle of *D*. *discoideum* may involve many other tradeoffs to be considered in the future, such as allocating more cells in the spore body at the expense of reducing the dispersal ability by creating a shorter stalk. Fourth, for simplicity, we have encapsulated here all the ecological variability in the starvation times; however, environmental heterogeneity may also come into play in other ways, e.g. in the way resources reappear in the system (progressively instead of instantaneously, or seasonally instead of throughout the year [15]) or in the amount of nutrients available at the beginning of each growth cycle, which here we have taken to be constant as a first approach. This could introduce additional tradeoffs arising from strains showing different feeding behavior [41]. Finally, our model does not include mutation, meiotic recombination or horizontal gene transfer, all of which can play an important role in producing diversity that could lead to coexistence, at least temporarily. Although mutation rates are very low in *D*. *discoideum* [42], meiotic recombination appears to occur at a sufficiently high rate that it could potentially influence population composition [43].

Our results show broadly that in fluctuating environments multicellularity and sociality are just part of a set of risk-management strategies. Although our work was motivated by *D*. *discoideum* in particular and by microbes in general, these results can be extended to other species where similar ecologically-induced tradeoffs between aggregation time, size and social behavior have been identified [44,45].

## Methods

### Implementation of the evolutionary simulations

To determine the winning genotype in each environment, we performed numerical simulations of several growth-starvation cycles in environments defined by their starvation time (for deterministic ones) or by their average starvation time (for stochastic ones). For the latter, starvation times were a stochastic variable exponentially distributed. The spectrum of genotypes was discretized, using 44772 genotypes for stochastic environments (1092 values of *α* and 41 values of *c*) and 4141 for deterministic environments (101 values of *α* and 41 values of *c*). The values of *c* range between *c* = 0.05 and *c* = 0.45 with a sampling of 10^−2^. In stochastic environments, the values of *α* were chosen with a sampling of 10^−4^ between *α* = 0 and *α* = 0.1 and with a sampling of 10^−2^ between *α* = 0.1 and *α* = 1. This irregular sampling was chosen to avoid abrupt jumps in the winning *c* in environments that select for strategies with a small investment in spores. In deterministic environments, however, the sampling in *α* was homogeneous, with a sampling step of 0.01 between *α* = 0 and *α* = 1.

In deterministic environments a single run was used (randomness introduced by the initial condition was tested and shown to be irrelevant in determining the stationary state) while in stochastic environments averages were taken over 20 independent realizations. In both cases simulations were run until *t* = 10^8^ hours and the winning genotype defined as the most abundant at that time. This was done for computational feasibility, longer simulations shown that in the long-term only one genotype survives. In stochastic environments the variance of this measurement is very low and the mean value coincides with the result of each single run. S1 Fig and S2 Fig show the short time evolution of some genotypes in deterministic and stochastic environments.

Initial abundances of each genotype were independently drawn from a standard log-normal distribution and subsequently normalized so that the entire population contained 10^8^ cells. An initial resource pulse of magnitude 10^8^ was added and the trajectories governed by Eq (1) was integrated using a Runge-Kutta numerical method until resources are exhausted (*R* reaches a zero value). The depletion of the resources triggers the onset of starvation and the population instantaneously splits between aggregators and non-aggregators and aggregators instantaneously differentiate between spores and stalk cells. A fraction *α* of cells aggregate and the remaining 1 - *α* remains as solitary (non-aggregated) cells. Finally, the population of aggregators is multiplied by a factor *s =* 0.8 that accounts for the spore:stalk cell differentiation.

The starvation onset is followed by a starvation phase in which both populations decline due to the death of spores and non-aggregated cells. To evaluate the final population size of both classes of cells at the end of the starvation phase, due to the fact that Eq (3) has an analytical solution, the functions are evaluated at *t* = *T* (if the environment is stochastic, this time is previously sorted from an exponential distribution), which significantly speeds up the simulations.

### Correlations between non-social traits

Simulations are stopped at the starvation onset, when resources have been exhausted and the population splits between aggregators and non-aggregators but before entering in the starvation phase in which cells and spores die.

### Chimeric success

Genotypes underwent one growth and after resources consumption, the populations split between a fraction *α* of aggregators and (*1-α*) of non-aggregators. Let $X_{\alpha ,c}^{sp}$ be the amount of spores produced by genotype (*α*, *c*), which is obtained by multiplying the number of aggregators by a factor *s = 0*.*8* to account for the stalk:spore cell differentiation. The pairwise chimeric success of genotype (*α*, *c*) against genotype (*α*, *c*′) is given by $X_{\alpha ,c}^{sp}/(X_{\alpha ,c}^{sp}+X_{\alpha^{\prime },c^{\prime }}^{sp})$ and the average chimeric success of genotype (*α*, *c*) is given by:

$$CS(\alpha ,c)=\frac{1}{N_{st}-1}\sum_{\alpha^{\prime },c^{\prime }}\frac{X_{\alpha ,c}^{sp}}{X_{\alpha ,c}^{sp}+X_{\alpha^{\prime },c^{\prime }}^{sp}}$$ (5)

where the sum runs over all the possible values of *α* and *c* except for *α* = *α*′ and *c = c*′ simultaneously and *N*_*st*_ is the total number of strains considered in the average.

### Linear hierarchies of chimeric dominance

To construct the linear hierarchies (Fig 4), the 31 winning genotypes obtained from Fig 2B are ordered according to the environment they evolved in. Label 0 corresponds to the winning genotype from the environment with *λ*_*T*_ = 10 hour and label 30 to the winning genotype that evolved in the environment with *λ*_*T*_ = 10^4^ hour. All strains are mixed in pairs, they undergo a growth-starvation cycle, and pairwise chimeric success is measured. To define a dominance / subordination relationship between the genotypes we set one of the genotypes in the mix as the reference genotype and its partner as the mixed genotype. If the reference genotype makes more spores than the mixed genotype, then it has a higher chimeric success, which is indicated by a red square in Fig 4. If the reference genotype makes fewer spores it will have a lower chimeric success (blue squares in Fig 4), and both genotypes will be equally successful if the number of spores is the same (gray squares in Fig 4).

## Supporting Information

S1 Text Continuous mechanism. Detailed description and analysis of the continuous aggregation mechanism.
(PDF)

S1 Table Description of the model parameters and their values. The lower part of the table gives the parameters that are allowed to evolve.
(DOCX)

S1 Fig Short-time evolution of populations in deterministic environments. A) Winning genotype in each environment, given by its investment in spores *α* (blue squares), and division rate *c* (red circles). Three environments are chosen and marked with a green circle, two of them select for *α =* 0 strategies and one for *α =* 1. B) Fast-recovery environment (*T =* 10 hour): fast reproducing strains have an advantage in the short run, but are in the long run outcompeted by the genotype with the lowest division rate. C) Intermediate-recovery environments (*T =* 158 hour): fast-reproducing strains are able to maintain their competitive advantage throughout. D) Slow-recovery environments (*T =* 199 hour): selection for genotypes with slow cell division.
(TIF)

S2 Fig Temporal evolution of populations in stochastic environments. A) Winning genotype in each environment, given by its investment in spores, *α* (blue squares), and division rate, *c* (red circles). Three environments are chosen and marked with a green circle. B,C) For intermediate environments (*λ*_*T*_ = 100 hour and *λ*_*T*_ = 358 hour respectively), populations show high amplitude fluctuations since the variation in the starvation times favors in each cycle a different genotype. D) For slow environments (*λ*_*T*_ = 1000 hour), the amplitude of the fluctuations in the population size decreases since most of the starvation times favor genotypes with *α* = 1.
(TIF)

S3 Fig Sensitivity analysis of the winning genotype for the choice of survival cost function, *β*. Increasing the survival probability of bigger cells over time (*β* = 5.1 − 8*c*) reduces selection for fast reproducing strains. The cost of small spores in terms of germination survival was not changed, so fast spore-selecting environments select for the same division rate as in Fig 2. Simulations are initialized with a number of genotypes that compete through several growth-starvation cycles. For computational feasibility the winner is determined as the most abundant genotype at *t =* 10^8^, when a few genotype still survive. Larger realizations show that only the winner is able to survive in the stationary state. A) Deterministic environments. B) Stochastic environments.
(TIF)

S4 Fig Sensitivity analysis for different choices of survival cost function *β* in deterministic environments. Modifying the tradeoff between cell survival and cell size leads to selection for different division rates in the *α* = 0 environments. A, B, C) The top row shows three families of curves where the survival advantage of bigger cells against the smaller ones increases from left to right. A) *β* = 3.1 − 4*c*, B) *β* = 5.5 − 10*c*, C) *β* = 19 − 40*c*. D) Selected division rate as a function of the starvation time in deterministic environments.
(TIF)

S5 Fig Sensitivity analysis for the initial condition and the Michaelis-Menten rates. A) The initial population size does not modify the winning genotype, only the transient dynamics. Highly diluted initial populations initially favor genotypes with a high division rate. However, once the total population reaches the carrying capacity, genotypes with a faster division rate start declining and eventually go to extinction. On the contrary, if the initial population is above the carrying capacity the total number of cells decreases at short times. Bigger cells take advantage of their longer survival, but as the population reaches the carrying capacity genotypes with a higher division rate start growing and finally outcompete the slower strains. Initial populations of each genotype were drawn from a log-normal distribution and the total population subsequently normalized to 10^3^, 10^7^, 10^8^ and 10^10^ cells. The size of the food pulse was kept constant, so increasing the population size increases the competition for resources. Data points are not shown for clarity, sampling in the mean starvation time as in panel B. B) Increasing the saturation constant *R*_*1/2*_ anticipates selection for completely aggregating strategies (*α* = 1) since the growth term decreases. Environments with a given mean starvation time become harsher and it is more beneficial to make more spores and reproduce faster. The initial population was fixed at 10^8^ cells and the amplitude of each food pulse at 10^8^. Squares indicate division rate and circles aggregator to non-aggregator ratio.
(TIF)

S6 Fig Tradeoff between number of spores and cell and spore size under different simulation settings. When the strains are plated on abundant resources and grow exponentially during a fixed time, followed by sudden starvation, A) the population size and B) the number of spores correlate positively with the division rate. Logarithmic scale used for the vertical axis in both the panels.
(TIF)
