# Correction: Lack of Ecological and Life History Context Can Create the Illusion of Social Interactions in *Dictyostelium discoideum*

**DOI:** 10.1371/journal.pcbi.1005850

**Published:** 2018-01-23

**Authors:** The PLOS Computational Biology Staff

There is an error in the second part of Eq 1 in the manuscript. The authors confirm that the errors affect the results in Fig 3 and S1 Text Figure C, but not in any of the other figures of the paper. Below are the details of the affected results:

The model in the original manuscript is cell size independent and therefore it does not include a tradeoff between cell size and division rate, as it is claimed in the text. For this, the equations need to be modified such that genotypes that produce bigger cells also consume more resources per division event. In the absence of any information on metabolic tradeoffs in slime molds, and given the lack of general trends for microbes, the simplest way to achieve this is to include a factor *Q* in the resource dynamics that indicates how the resource needed to produce a single consumer cell scales with cell size. We choose *Q = c*_*max*_*/c* to be equal to 1 for the fastest dividing strain, which produces the smallest cells; *Q* then increases as the division rate decreases (cell size increases). Thus, all strains consume resources at the same rate *c*_*max*_ but some wait longer between cell divisions than others. The corrected resource dynamics in Eqs (1) is

$$\dot{R}=-\frac{R}{R_{1/2}+R}\sum_{*,c}QcX_{*,c}$$

The inclusion of this additional tradeoff only changes the carrying capacity of the system and does not modify the outcome of the competition among strains; therefore, this correction does not change the results of the evolutionary simulations (Fig 2, S3-S5 Fig) or of the simulations that involve mixes (Figs 4 and 5). S6 Fig does not change either, because it is independent of resource dynamics. The only effects appear in S1, S2 Fig in the final population sizes but not in the observed trends, and in Fig 3 and S1 Text Fig C, which show the correlations between non-social traits for the discrete, respectively the continuous mechanism. Since bigger cells need more resources per division event and since the amount of resources used in the growth period is fixed across genotypes, strains with lower division rate (bigger cells) produce smaller populations upon resource exhaustion (corrected Fig 3A below).

For the discrete mechanism, this result leaves the majority of correlations between non-social traits unchanged, except those for genotypes that differ in *c* but not in *α*: i.e. slow environment genotypes for which all cells aggregate (i.e. no non-aggregators, *α = 1*) or fast environment genotypes for which all cells stay solitary (i.e. no aggregators, *α = 0*). Despite having the same *α*, due to the cell size–division rate tradeoff, these genotypes differ in the production of aggregating (Fig 3B below), respectively non-aggregating cells (Fig 3E below),and these cell numbers correlate positively with *c* (Fig 3C and 3F below).

For the continuous mechanism, prior to the correction, strains with the same aggregation rate *γ* but higher division rate *c* produced fewer aggregated cells, since cell production upon resource exhaustion was independent of *c* (original Fig 3A) and faster dividing non-aggregators had lower survival and were thus less likely to aggregate before dying. However, when the cell size versus division rate tradeoff is included correctly, strains with higher division rates produce more cells upon resource exhaustion (Fig 3A below), which, given our choice for the functional dependence of the tradeoffs, counters the effect of the tradeoff between division rate and short-term survival in the number of both aggregators and non-aggregators. Consequently, correlations between number of aggregators (respectively non-aggregators) and *c* and *γ* for slow and fast environment genotypes change (see corrected S1 Text Figure C below) and they become qualitatively similar to the ones obtained for the discrete mechanism (below in Fig 3 and S1 Text Figure C compare: 3B with C (i); 3C with C (ii); 3D with C (vi); 3E with C (iv) and 3F with C (v)). The correlation between number of aggregators and aggregation time also switches from positive to negative in slow environment genotypes (S1 Text Fig C (iii)).

Please view the correct versions of Fig 3 and S1 Text Figure C here:

**Fig 3:**
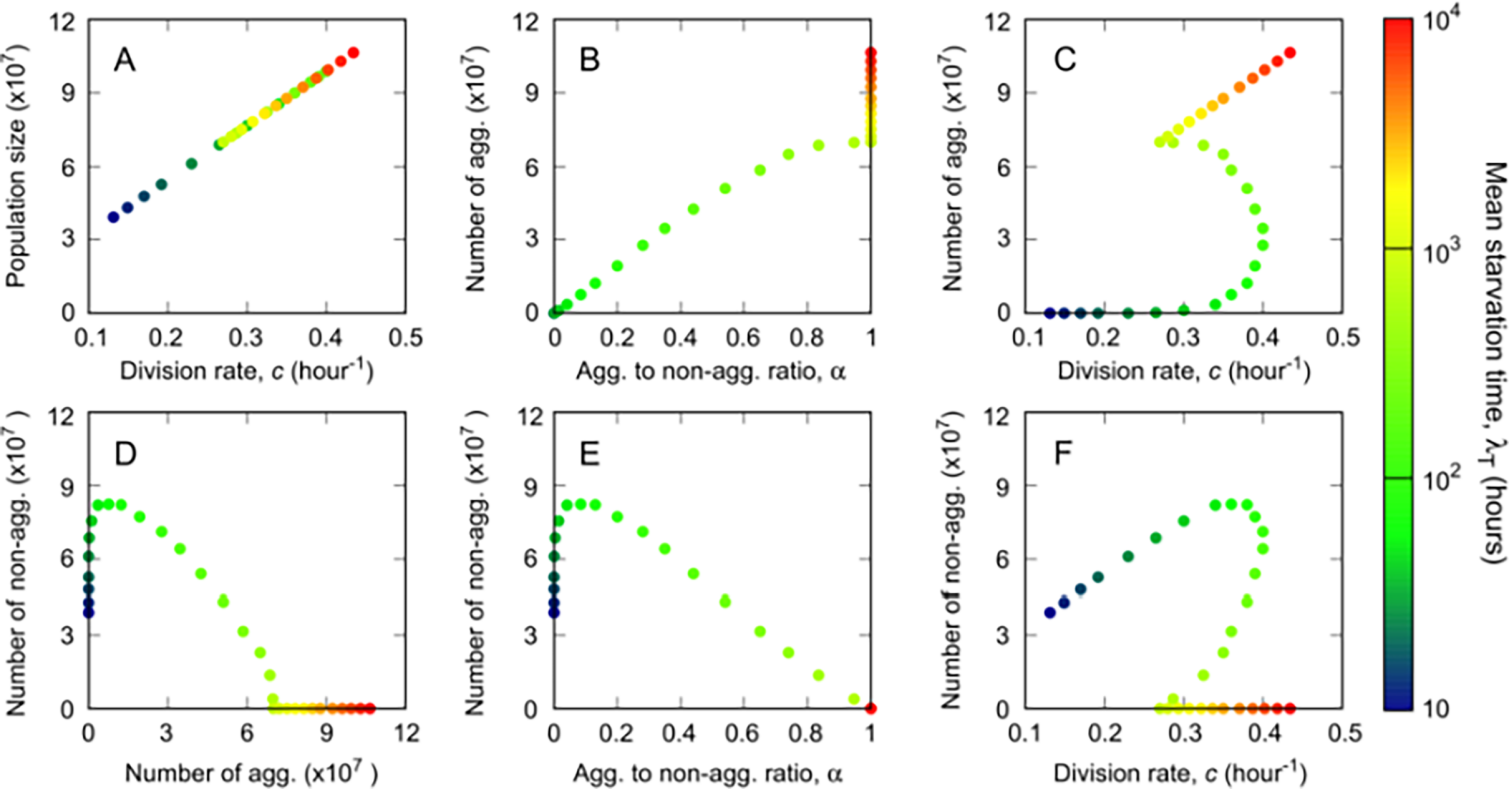
Correlations between non-social traits. A clonal growth period for each one of the 31 winning genotypes obtained in Fig 2 is integrated to evaluate correlations between the non-social traits included in the model at the onset of starvation. A) Total population versus division rate, *c*. Number of aggregators versus B) the aggregator to non-aggregator ratio, *α*; C) division rate, *c*. Number of non-aggregators versus D) the number of aggregators; E) the aggregator to non- aggregator ratio; F) the division rate.

## Supporting information

S1 Text Figure C. Correlations between non-social traits. Each one of the 31 winning genotypes obtained in S1 Text Fig B (ii) is grown clonally. Top row: aggregator-related traits are measured after a growth-starvation sequence with a starvation time of 200 hours. The number of aggregators is related to: A) aggregation rate, B) division rate and C) aggregation time. Bottom row: non-aggregator related traits are measured after 160 hours of starvation, when the genotypes with the lowest aggregation rate have produced 95% of their final number of aggregators. The number of solitary cells is related to D) aggregation rate, E) division rate, and F) number of aggregators.
(DOCX)
