# Supplementary material for: Lack of Ecological and Life History Context Can Create the Illusion of Social Interactions in Dictyostelium discoideum
Source: PLoS Comput Biol. 2016 Dec 15;12(12):e1005246. doi: 10.1371/journal.pcbi.1005246 (PMC5157950; doi:10.1371/journal.pcbi.1005246)
Supplement: S1 Table — The lower part of the table gives the parameters that are allowed to evolve. (DOCX) [file pcbi.1005246.s002.docx]

| **Description** | **Parameter** | **Value** | **Units** |
| --- | --- | --- | --- |
| Rate of change of the vegetative cell death rate | *μ* | 2 x 10^-3^ | hour^-1^ |
| Maximum lifetime of a vegetative cell | *T_sur_* | 200 | hour |
| Spore germination time | *τ* | 4 | hour |
| Spore mortality rate | *δ* | 2 x 10^-4^ | hour^-1^ |
| Fraction of spores within the aggregate | *s* | 0.8 | --- |
| Initial food pulse | *R_0_* | 10 ^8^ | # cells |
| Half-saturation constant of resources consumption | *R_1/2_* | 0.1R_0_ | # cells |
| Division rate | *c* | Varied | hour^-1^ |
| Aggregator to non-aggregator ratio (discrete) | *α* | Varied | --- |
| Aggregation rate (continuous) | *γ* | Varied | hour ^-1^ |
