# Supplementary figures and images for: Lack of Ecological and Life History Context Can Create the Illusion of Social Interactions in Dictyostelium discoideum
Source: PLoS Comput Biol. 2016 Dec 15;12(12):e1005246. doi: 10.1371/journal.pcbi.1005246 (PMC5157950; doi:10.1371/journal.pcbi.1005246)

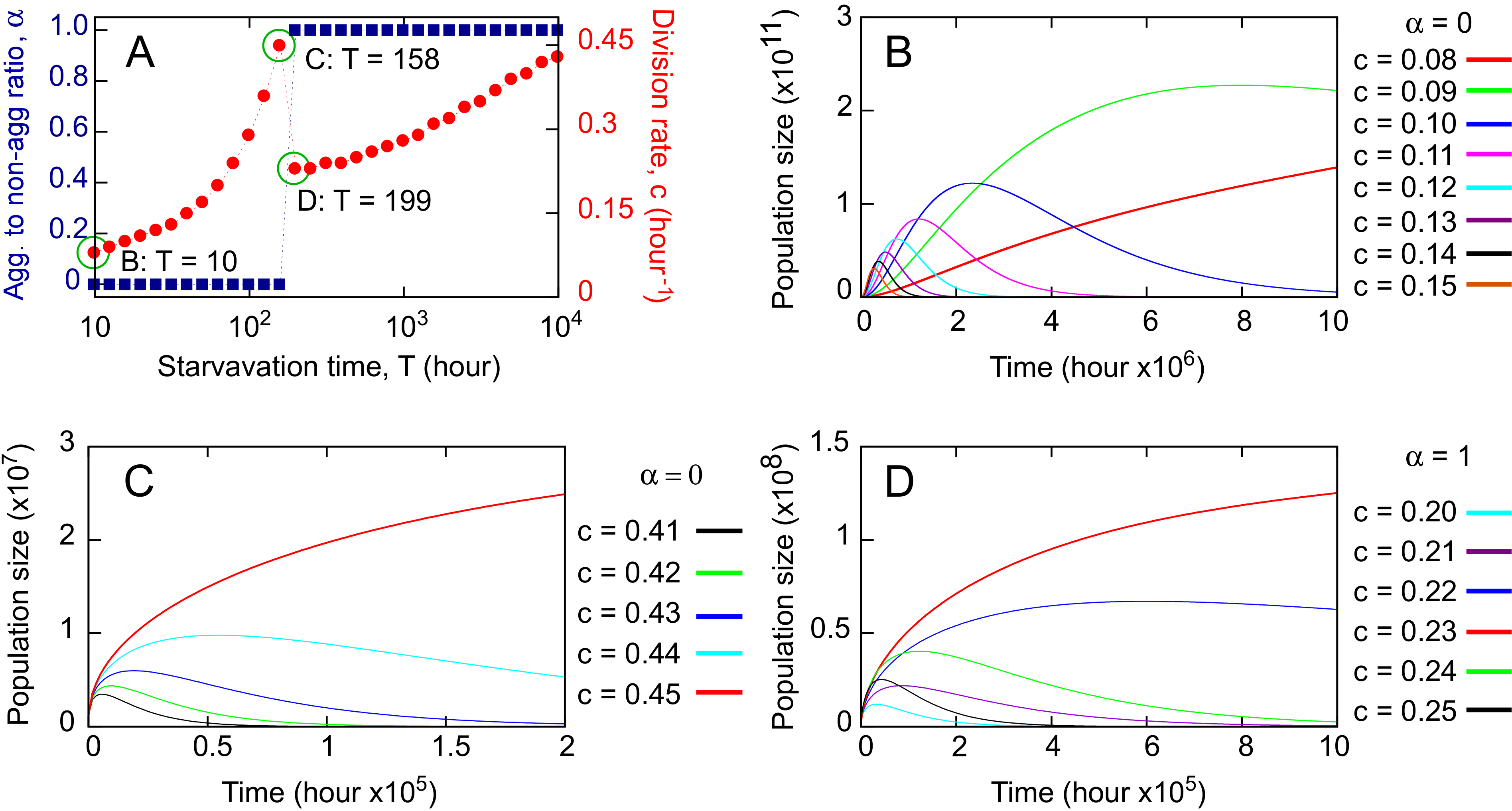

Supplement: S1 Fig — A) Winning genotype in each environment, given by its investment in spores α (blue squares), and division rate c (red circles). Three environments are chosen and marked with a green circle, two of them select for α = 0 strategies and one for α = 1. B) Fast-recovery environment (T = 10 hour): fast reproducing strains have an advantage in the short run, but are in the long run outcompeted by the genotype with the lowest division rate. C) Intermediate-recovery environments (T = 158 hour): fast-reproducing strains are able to maintain their competitive advantage throughout. D) Slow-recovery environments (T = 199 hour): selection for genotypes with slow cell division. (TIF) [file pcbi.1005246.s003.tif]

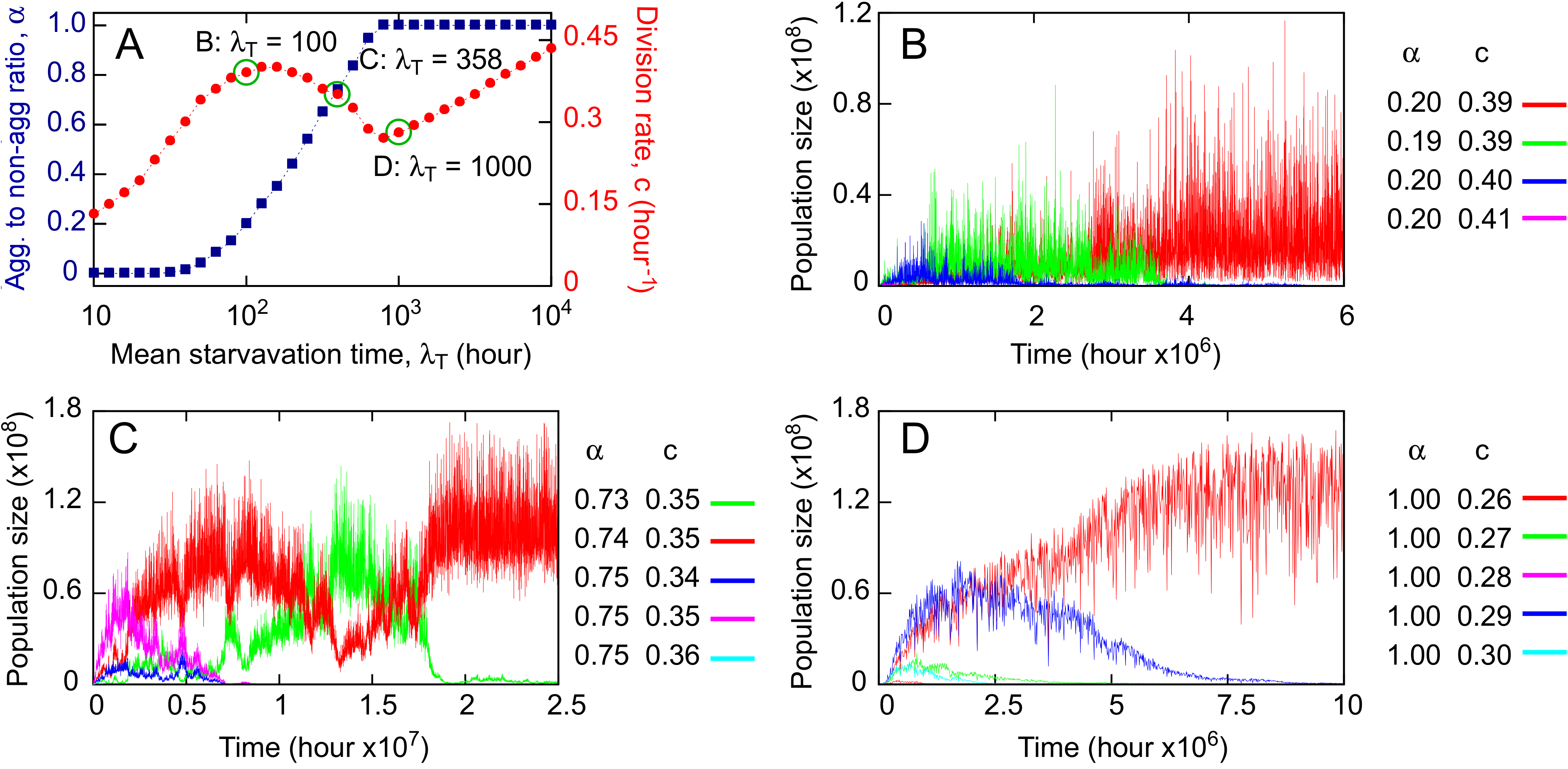

Supplement: S2 Fig — A) Winning genotype in each environment, given by its investment in spores, α (blue squares), and division rate, c (red circles). Three environments are chosen and marked with a green circle. B,C) For intermediate environments (λT = 100 hour and λT = 358 hour respectively), populations show high amplitude fluctuations since the variation in the starvation times favors in each cycle a different genotype. D) For slow environments (λT = 1000 hour), the amplitude of the fluctuations in the population size decreases since most of the starvation times favor genotypes with α = 1. (TIF) [file pcbi.1005246.s004.tif]

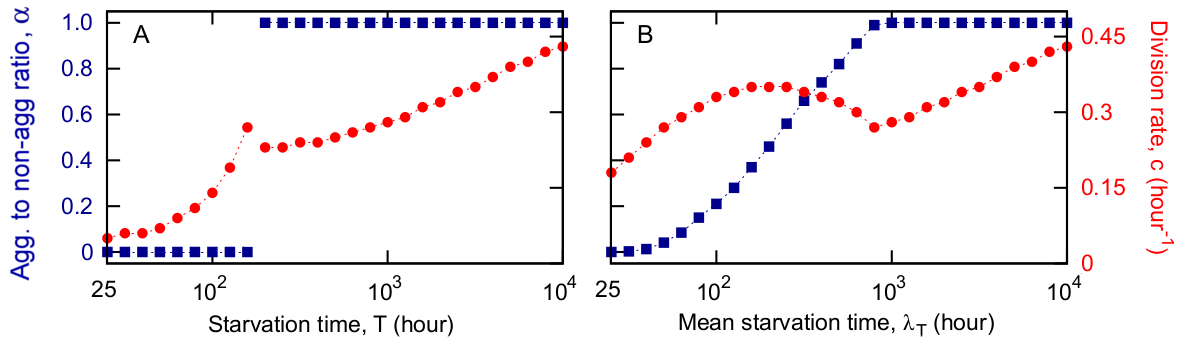

Supplement: S3 Fig — Increasing the survival probability of bigger cells over time (β = 5.1 − 8c) reduces selection for fast reproducing strains. The cost of small spores in terms of germination survival was not changed, so fast spore-selecting environments select for the same division rate as in Fig 2. Simulations are initialized with a number of genotypes that compete through several growth-starvation cycles. For computational feasibility the winner is determined as the most abundant genotype at t = 108, when a few genotype still survive. Larger realizations show that only the winner is able to survive in the stationary state. A) Deterministic environments. B) Stochastic environments. (TIF) [file pcbi.1005246.s005.tif]

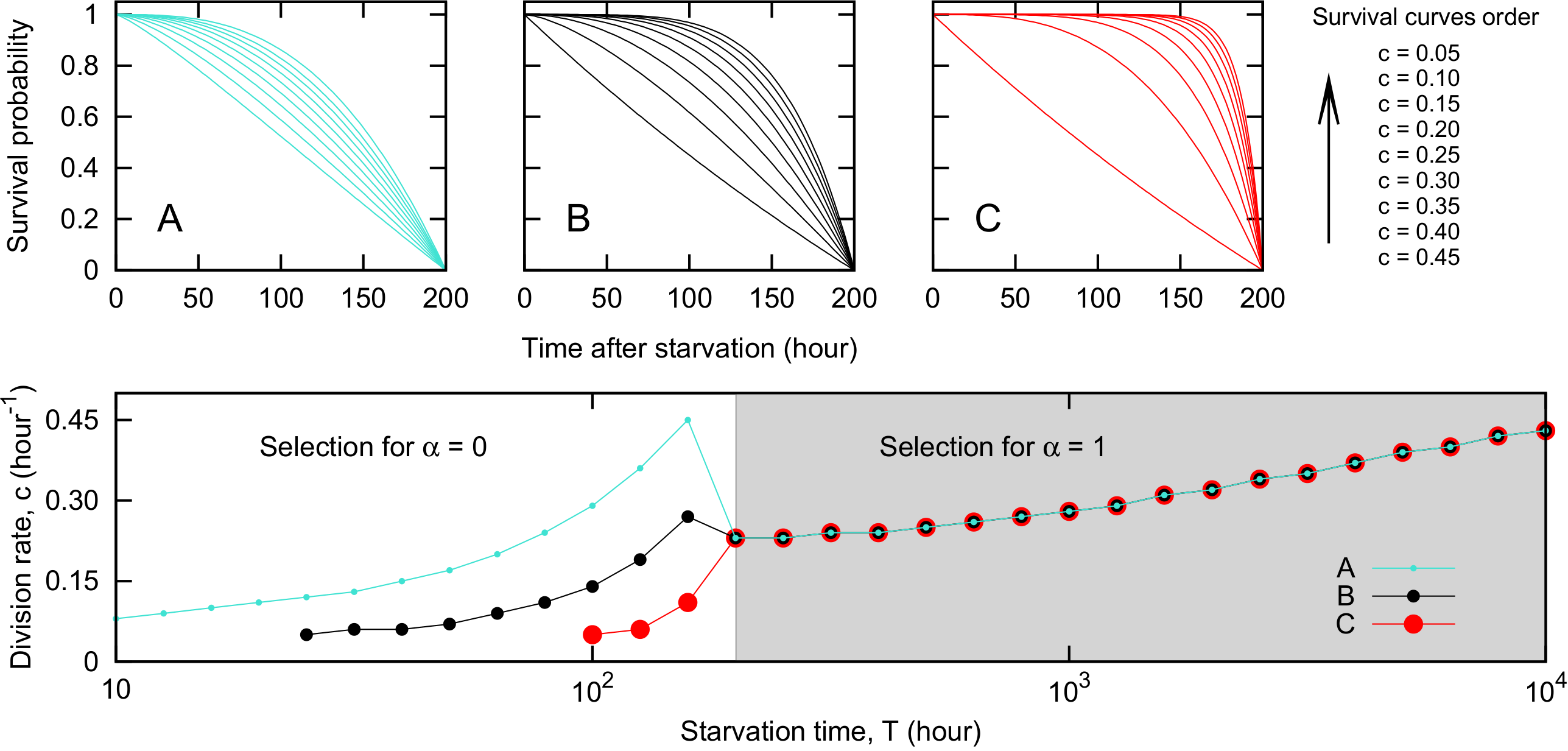

Supplement: S4 Fig — Modifying the tradeoff between cell survival and cell size leads to selection for different division rates in the α = 0 environments. A, B, C) The top row shows three families of curves where the survival advantage of bigger cells against the smaller ones increases from left to right. A) β = 3.1 − 4c, B) β = 5.5 − 10c, C) β = 19 − 40c. D) Selected division rate as a function of the starvation time in deterministic environments. (TIF) [file pcbi.1005246.s006.tif]

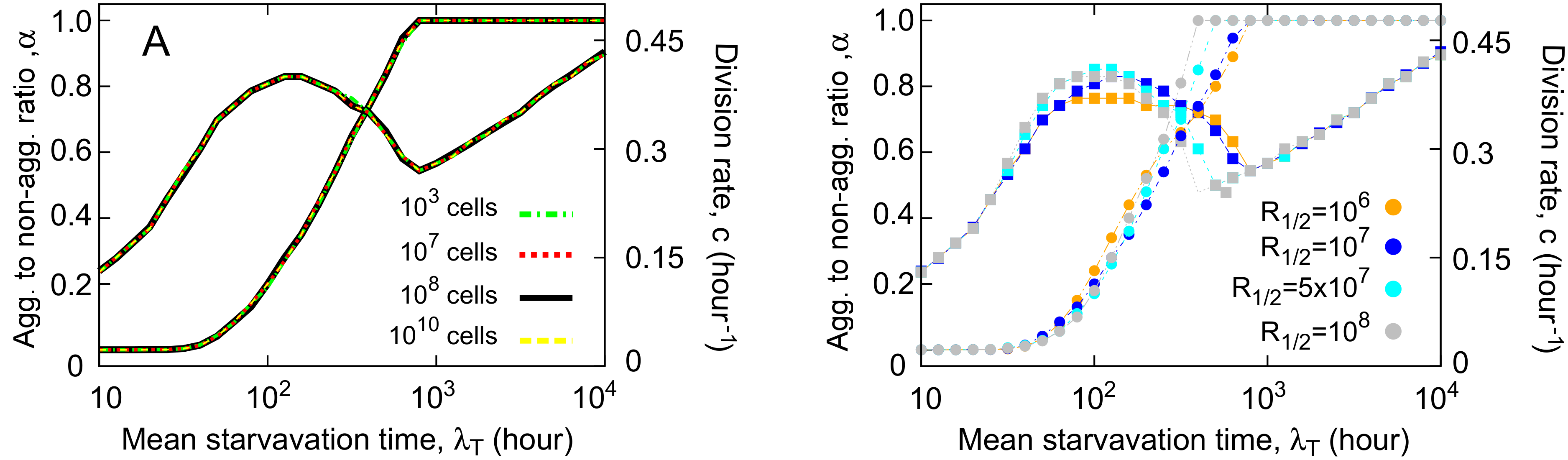

Supplement: S5 Fig — A) The initial population size does not modify the winning genotype, only the transient dynamics. Highly diluted initial populations initially favor genotypes with a high division rate. However, once the total population reaches the carrying capacity, genotypes with a faster division rate start declining and eventually go to extinction. On the contrary, if the initial population is above the carrying capacity the total number of cells decreases at short times. Bigger cells take advantage of their longer survival, but as the population reaches the carrying capacity genotypes with a higher division rate start growing and finally outcompete the slower strains. Initial populations of each genotype were drawn from a log-normal distribution and the total population subsequently normalized to 103, 107, 108 and 1010 cells. The size of the food pulse was kept constant, so increasing the population size increases the competition for resources. Data points are not shown for clarity, sampling in the mean starvation time as in panel B. B) Increasing the saturation constant R1/2 anticipates selection for completely aggregating strategies (α = 1) since the growth term decreases. Environments with a given mean starvation time become harsher and it is more beneficial to make more spores and reproduce faster. The initial population was fixed at 108 cells and the amplitude of each food pulse at 108. Squares indicate division rate and circles aggregator to non-aggregator ratio. (TIF) [file pcbi.1005246.s007.tif]

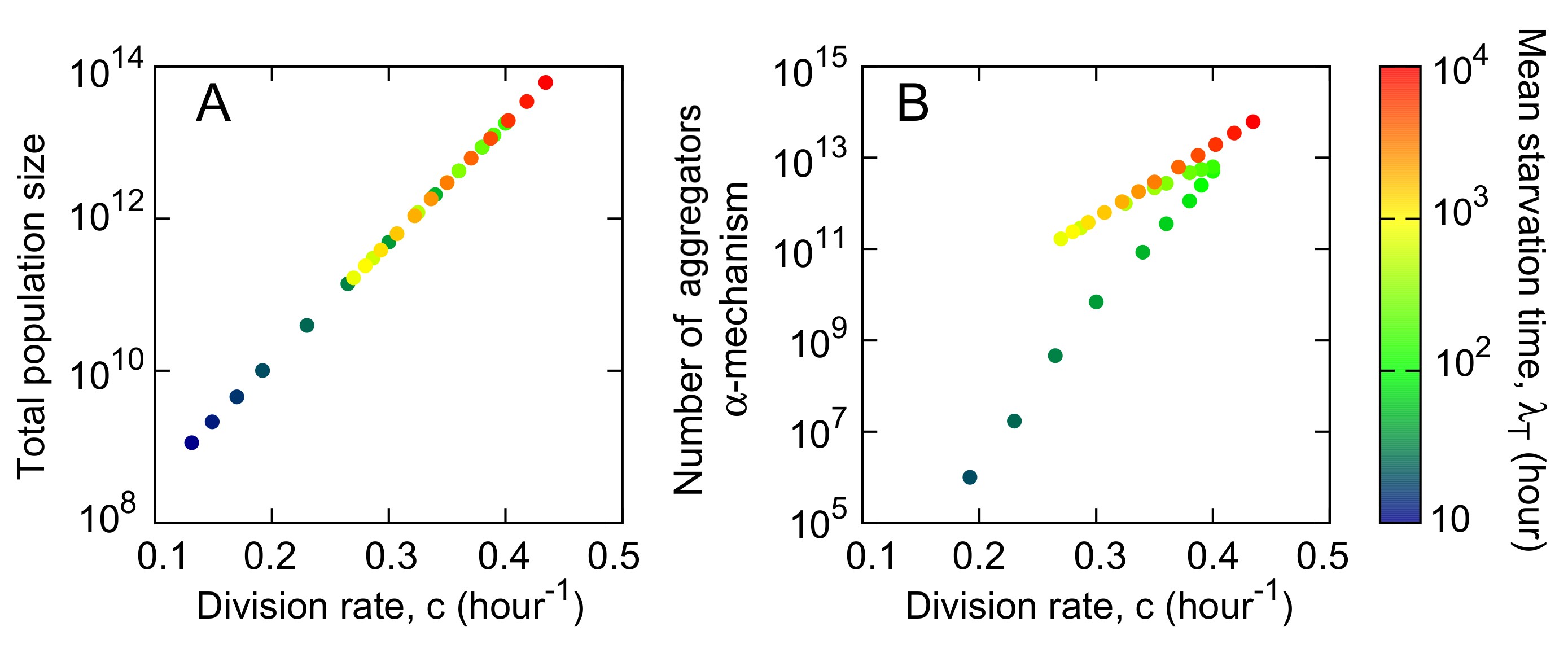

Supplement: S6 Fig — When the strains are plated on abundant resources and grow exponentially during a fixed time, followed by sudden starvation, A) the population size and B) the number of spores correlate positively with the division rate. Logarithmic scale used for the vertical axis in both the panels. (TIF) [file pcbi.1005246.s008.tif]
