# Supplementary material for: Correction: Lack of Ecological and Life History Context Can Create the Illusion of Social Interactions in Dictyostelium discoideum
Source: PLoS Comput Biol. 2018 Jan 23;14(1):e1005850. doi: 10.1371/journal.pcbi.1005850 (PMC5779642; doi:10.1371/journal.pcbi.1005850)
Supplement: S1 Text — Each one of the 31 winning genotypes obtained in S1 Text Fig B (ii) is grown clonally. Top row: aggregator-related traits are measured after a growth-starvation sequence with a starvation time of 200 hours. The number of aggregators is related to: A) aggregation rate, B) division rate and C) aggregation time. Bottom row: non-aggregator related traits are measured after 160 hours of starvation, when the genotypes with the lowest aggregation rate have produced 95% of their final number of aggregators. The number of solitary cells is related to D) aggregation rate, E) division rate, and F) number of aggregators. (DOCX) [file pcbi.1005850.s001.docx]

**Supporting information for**

**Lack of Ecological and Life History Context Can Create the Illusion of Social Interactions in *Dictyostelium discoideum***

**Ricardo Martinez-Garcia^1^ & Corina E. Tarnita^1^**

**^1^ Department of Ecology and Evolutionary Biology,**

**Princeton University, Princeton NJ, USA**

**S1 Text. Continuous aggregation mechanism (phenotypic variation).**

In the main text we focus on a discrete mechanism for the formation of the aggregate in which starvation triggers an instantaneous population partitioning between aggregators and non-aggregators followed by a spore:stalk cell differentiation within the aggregators. The investment in each type of cells is characteristic of each genotype and determined by the aggregator to non-aggregator ratio *α*. In this appendix, we analyze a different mechanism by which starving cells turn into aggregators continuously in time at an aggregation rate, *γ*, that is characteristic of each genotype. The main difference between these two mechanisms is that while in the discrete approach only a certain fraction of cells can join the aggregate and the others remain solitary, in the continuous approach every cell has the potential to aggregate but some take longer than others (Fig A). Therefore, this latter approach also allows the quantification of an aggregation time, defined as the time at which all the solitary cells in a starving population either turn into aggregators or die. Non-aggregators can still be left behind, but only if the starvation period is shorter than the aggregation time. Therefore, the number of non-aggregators that a given genotype (*γ, c*) leaves behind depends on the length of the starvation interval: the shorter the starvation period, the more non-aggregators will be left behind.

For simplicity, we assume that non-aggregators and aggregators/spores (depending on whether cell differentiation has already taken place or not) die continuously during the entire starvation phase. Aggregation occurs continuously until the end of the starvation phase or until all cells are either in an aggregate or dead. Aggregation is followed by cell differentiation between spores and stalk. The equations for the dynamics of each subpopulation are different from Eq. (3) in the main text, which show the dynamics of spores and non-aggregators for the discrete case. While aggregation is happening (i.e. $X_{\gamma,c}\neq0$) the equations include an additional term that accounts for the continuous transfer of cells from non-aggregators to aggregators

$\dot{X}_{\gamma,c}^{ag}\left( t \right)=-\delta X_{\gamma,c}^{ag}\left( t \right)+\gamma X_{\gamma,c}$

$\dot{X}_{\gamma,c}=\tilde{X}_{\gamma,c}\dot{S_{c}}\left( t \right)-\gamma X_{\gamma,c}$ (S1)

Then, once there are no more solitary cells (or at the end of the starvation time), the number of spores is obtained by multiplying the population of aggregators by a factor *s=*0.8 that accounts for the spore:stalk differentiation. If the starvation time is larger than the aggregation time, spores continue dying until the end of the starvation time

$\dot{X}_{\gamma,c}^{sp}\left( t \right)=-\delta X_{\gamma,c}^{sp}\left( t \right)$ (S2)

Therefore, the final fraction of spores will be determined by the aggregation rate, the duration of the starvation phase (starvation time), and the survival of non-aggregators (which in our model is related to the division rate).

**Results**

**Evolution. Winning genotypes.** Running competition simulations like the ones described in the main text for the discrete aggregation mechanism, we obtain the winning genotypes (*γ, c*) for different environments. As a function of changing environmental conditions defined by the mean starvation time, both traits, *c* and *γ*, exhibit the same trend that was obtained for *c* and *α* in the discrete mechanism (Fig B).

**Correlations between non-social traits.** We determine the correlations between non-social traits (number of aggregators, number of non-aggregators, division rate, aggregation rate, and time to complete aggregation, henceforth aggregation time) using the winning genotypes from the different stochastic environments (Fig. B(ii)), as we did in the main text for the discrete mechanism. The only difference is that now it takes a finite but non-zero time to turn a population of starving cells entirely into aggregators and subsequently spores. This time depends on both the aggregation rate *γ* and the division rate *c*. If aggregation starts from a fixed number of starving cells, for fixed *c* the aggregation time decreases with increasing *γ*, since the higher *γ* is, the faster the aggregate gets formed; for fixed *γ* it decreases with increasing *c*, since the higher the division rate is, the faster the non-aggregators die leaving only the aggregators. If cells complete a full growth-starvation period, strains with higher division rate will initiate the aggregation from a larger population of starving cells (Fig. 3A in the C orrigendum), however, due to our particular choice for the functional dependence of the tradeoffs, the effect of the tradeoff between division rate and survival is more important on the aggregation times, which remains therefore inversely proportional to *c* for fixed *γ.*

The existence of a finite aggregation time establishes an important difference with the discrete mechanism, in which aggregation and cell differentiation take place immediately after food depletion and all quantities can be measured at that time. We therefore quantify aggregator-related traits (Fig C, top row) after a standardized time (same for all genotypes), picked to ensure that all genotypes have completed aggregation and there are no non-aggregators left in the system. The time used is *T_sur_* = 200 hours of starvation, which is the maximum amount of time that a starving solitary cell can live in our model. However, precisely because at this time there are no non-aggregators left, non-aggregator-related traits have to be quantified at some intermediate point at which at least some genotypes still have some non-aggregators (Fig C, bottom row). For this purpose, here we look at time *T =* 160 hours but the results hold for any starvation time *T* prior to complete aggregation.

In the simulation, we initialize clonal populations of the same size for each genotype, allow them to complete the growth-starvation sequence (with a starvation time of either 200 or 160 hours depending on whether we are interested in measuring aggregators or non-aggregators respectively) and measure correlations with the non-social traits enumerated above.

First of all, we will focus on the correlations between production of aggregators and the non-social traits: aggregation rate, division rate and aggregation time. If we fix *c*, the genotypes with higher *γ* have a higher number of aggregators since solitary cells aggregate faster and are less likely to die due to starvation. If we fix *γ*, the genotypes with higher *c* have a higher number of aggregators since they produce a larger population of starving cells that cancels the effect of their non-aggregators surviving shorter and being therefore more likely to eventually die before aggregating. However, our winning genotypes vary with both *c* and *γ,* and therefore *γ* and *c* interact to determine the number of aggregators. For intermediate-environment winning genotypes, *γ* increases and *c* decreases simultaneously (Fig B(ii)) and therefore they have opposite effects on spore number; their net effect, however, is dominated by *γ* so that for intermediate-environment winning genotypes the amount of aggregators increases with increasing *γ* (Fig, C(i)) and decreasing *c* (Fig. C(ii))*.* For slow-environment winning genotypes, *γ* is constant but *c* increases (Fig B(ii)), and therefore the number of aggregators increases (Fig C(i) and (ii)). For fast-environment winning genotypes both *γ* and *c* (Fig B(ii)) increase so that they enhance each other’s positive effect on the number of aggregators (Fig C(i) and (ii)). Aggregation time is inversely proportional to the aggregation rate and to the division rate; therefore, the number of aggregators decreases when aggregation time increases (Fig C(iii)). Second, we related the number of non-aggregators to division rate, aggregation rate and number of aggregators at an intermediate time at which at least some genotypes still had some non-aggregators. For intermediate-environment winning genotypes, we found that the number of non-aggregators decreases with aggregation rate *γ*, with the division rate *c* and with the number of aggregators (Fig C(iv), (v) and (vi)). For fast-environment winning genotypes, the effect of increasing *c* dominates the effect of increasing *γ* and the number of non-aggregators increases with aggregation rate, division rate and number of aggregators (Fig. C(v) and (vi)). For slow-environment winning genotypes, there are no non-aggregators at the intermediate time sampled.

It is important to note that the correlations between number of aggregators (respectively non-aggregators) and *c* and *γ* for slow and fast environment genotypes are qualitatively similar to the correlations obtained for the discrete mechanism (in Corrigendum Figure 3 and in S1 Text Figure C compare: 3B with C (i); 3C with C (ii); 3D with C (vi); 3E with C (iv) and 3F with C (v)). The correlation between number of aggregators and aggregation time also switches from positive to negative in slow environment genotypes (S1 Text Fig. C (iii)).

**Correlations between chimeric success and non-social traits.** To determine chimeric success for the continuous mechanism we simulate a single growth-starvation cycle in which we fix the starvation time at *T_sur_* = 200 hours since chimeric success is a spores-related quantity. Due to the fact that there will be no non-aggregators left at this time, this prevents us from exploring the correlation between chimeric success and the number of non-aggregators. Instead, we will measure how chimeric success correlates with the division rate, the average production of aggregators (similarly to what was done in the main text for the discrete mechanism) and the aggregation time.

The simulations are initialized with a population of size *X_0_* consisting of a 50:50 mix of two of the winning genotypes (Fig B(ii)). The size of the pulse of resources, *R_0_,* is kept constant and the initial number of cells varied to cover different initial cell densities relative to resource magnitude.

The results are mostly qualitatively similar to those obtained for the discrete mechanism: we find the same correlations between chimeric success and the average number of aggregators regardless of the initial density of cells (Fig D(ii), (v) and (viii)) and between chimeric success and division rate for low and intermediate initial densities (Fig D(i) and (iv)). At high initial densities, however, the correlation for slow-environment genotypes changes if aggregation is continuous (Fig D(vii)). Since chimeric success is measured after 200 hours of starvation, cells with a higher division rate also pay a higher survival cost. This results in a smaller population of aggregators (more starving cells die before aggregating) when increasing *c* and as a consequence, in a reduced chimeric success.

Finally, at low initial densities, chimeric success has a negative correlation with aggregation time (positive correlation with the aggregation rate) for fast- and slow-environment genotypes. However, for intermediate-environment genotypes the effect of the division rate dominates the effect of the aggregation rate and chimeric success and aggregation time show a positive correlation (Fig D(iii)). As the initial density of cells is increased, the growth period is shorter and thus the effect of the aggregation rate starts balancing that of the division rate. In the limit in which the initial density of cells is high, the growth phase is residual. Therefore, chimeric success correlates negatively with aggregation time over the whole set of genotypes (Fig D(vi) and (ix)).

**Methods**

**Simulation details.** During the growth period the equations are equivalent to Eq. (1) presented for the discrete aggregation mechanism in the main text. During the starvation phase, the new terms in Eq. (S1), corresponding to the continuous formation of aggregators, make the dynamics analytically intractable and this mechanism computationally heavier than the phenotypic switch (discrete mechanism) presented in the main text. Therefore, only 99 genotypes were used: 11 values of *γ* (*γ* = 0 and 10 additional values in the interval [10^-4^,10^-1^] given by *γ_i_* = 10^(^*^i^*^-13)/3^, equidistant in a logarithmic scale) and 9 values of *c* uniformly distributed in the interval [0.05, 0.45]. The dynamics in the growth and starvation periods is solved using a Runge-Kutta algorithm to numerically integrate Eq. (1) and Eq. (S1) and (S2) respectively.

**Aggregation time** (Fig A). To determine the speed at which a genotype *γ* completes its aggregate, clonal populations of the same initial size are initialized without food. In response to starvation, solitary cells turn into aggregators at rate *γ* and die following the survival curve of their respective genotype. Aggregators die at rate *δ*. The simulations are stopped after a starvation time *T_sur_* = 200 hours, when all the solitary cells have either died or have turned into aggregators. Finally, the number of aggregators at every time is normalized by its final number to obtain the rate at which this maximum value is reached.

**Chimeric success.** Genotypes underwent one growth-starvation cycle with a starvation time of 200 hours. During that time, starving solitary cells turn into aggregators at aggregation rate *γ* characteristic of each genotype. When the population of non-aggregators is exhausted (all the cells have either died or aggregated), the amount of spores $X_{\gamma,c}^{sp}$ produced by genotype (*γ, c*), is obtained by multiplying the number of aggregators at that time, $X_{\gamma,c}^{ag}$, by a factor *s* = 0.8 that accounts for the spores:stalk cell differentiation. Then, spores die until the fixed starvation time of 200 hours is reached. Finally, the chimeric success is obtained using Eq. (5) in the main text but replacing *α* with *γ*.


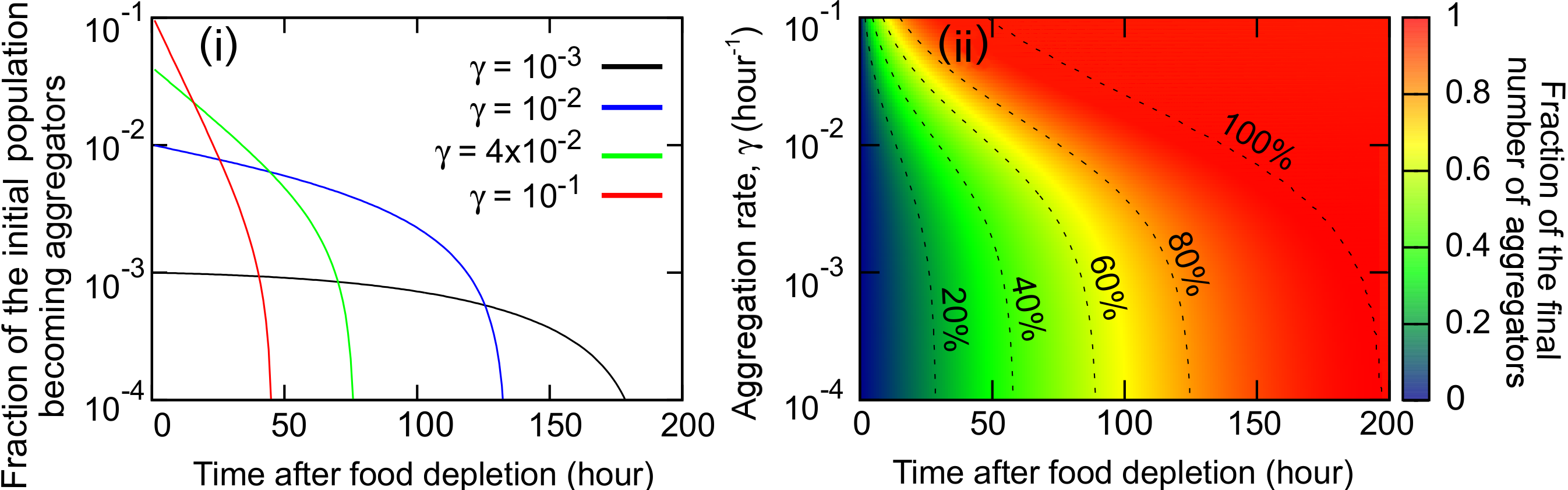


**Fig. A The continuous aggregation mechanism allows the study of aggregation time.** The division rate is kept constant in both panels *c =* 0.15. A) Starving cells of a given genotype aggregate at rate *γ*. Genotypes with higher *γ* make most of the aggregate immediately; genotypes with lower *γ* take longer to produce the aggregate. B) The speed of aggregate formation for a given *γ*. The aggregation time is indicated by the 100% contour line.


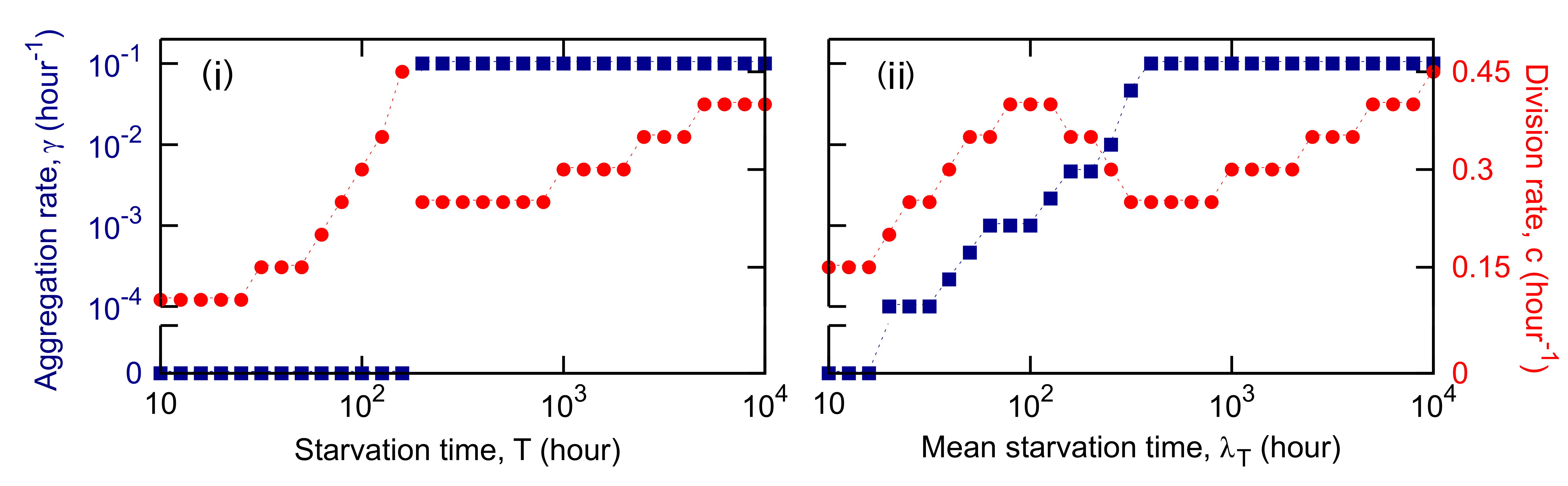


**Fig. B Winning genotypes in deterministic and stochastic environments.** Blue squares represent the aggregation rate and red circles represent the division rate, dashed lines are interpolations. Simulations are initialized with 99 genotypes (9 values of *c* and 11 values of *γ*) that compete through several growth-starvation cycles. For computational feasibility the winner is determined as the most abundant genotype at *t =* 10^8^, when a few genotypes still survive. Larger realizations show that only the winner is able to survive in the stationary state. A) Deterministic environments. B) Stochastic environments with exponentially distributed starvation times; averages taken over 20 independent realizations. Parameters as in S1 Table.


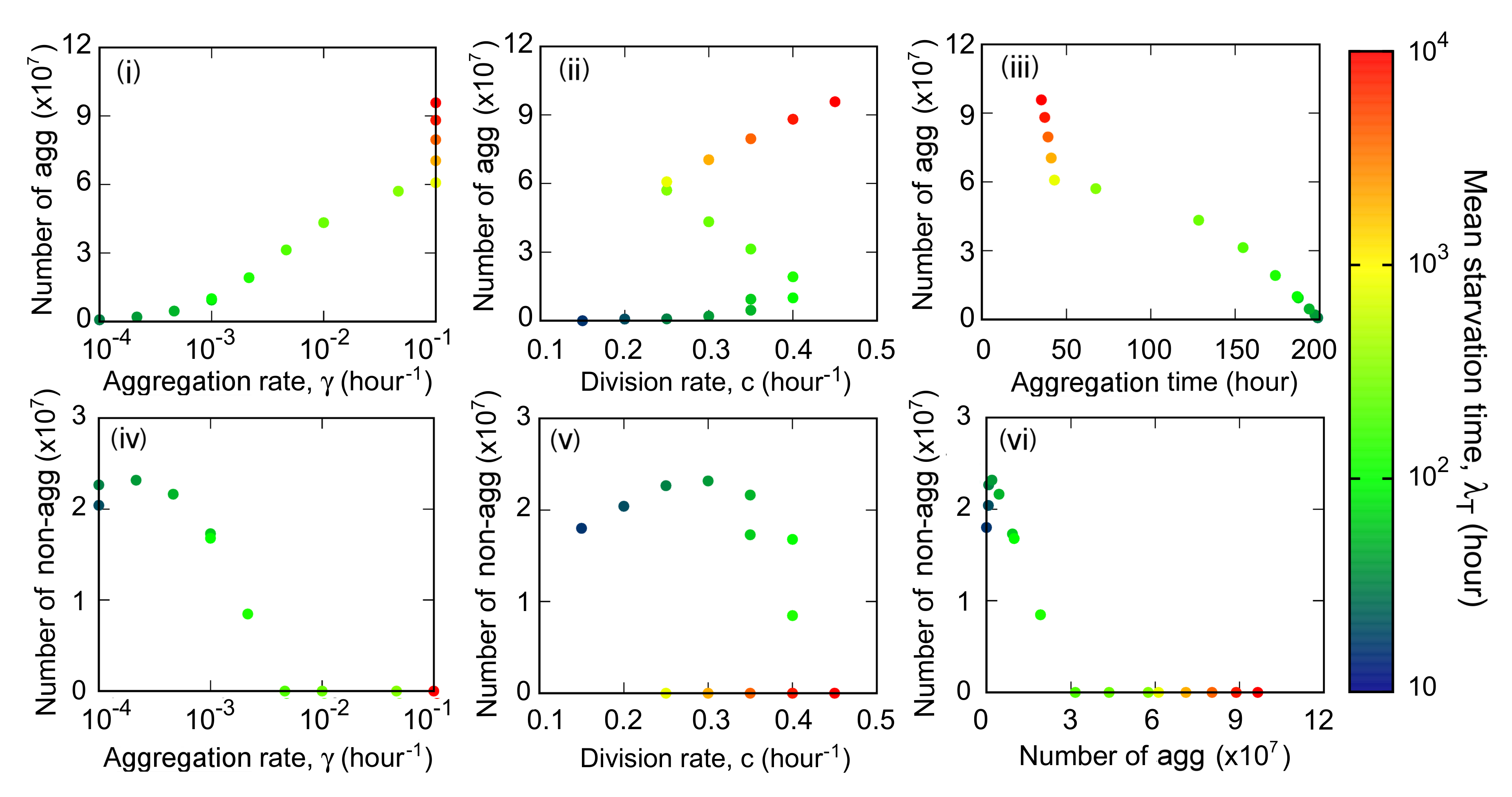


**Fig. C Correlations between non-social traits.** Each one of the 31 winning genotypes obtained in S1 Text Fig B (ii) is grown clonally. Top row: aggregator-related traits are measured after a growth-starvation sequence with a starvation time of 200 hours. The number of aggregators is related to: A) aggregation rate, B) division rate and C) aggregation time. Bottom row: non-aggregator related traits are measured after 160 hours of starvation, when the genotypes with the lowest aggregation rate have produced 95% of their final number of aggregators. The number of solitary cells is related to D) aggregation rate, E) division rate, and F) number of aggregators.


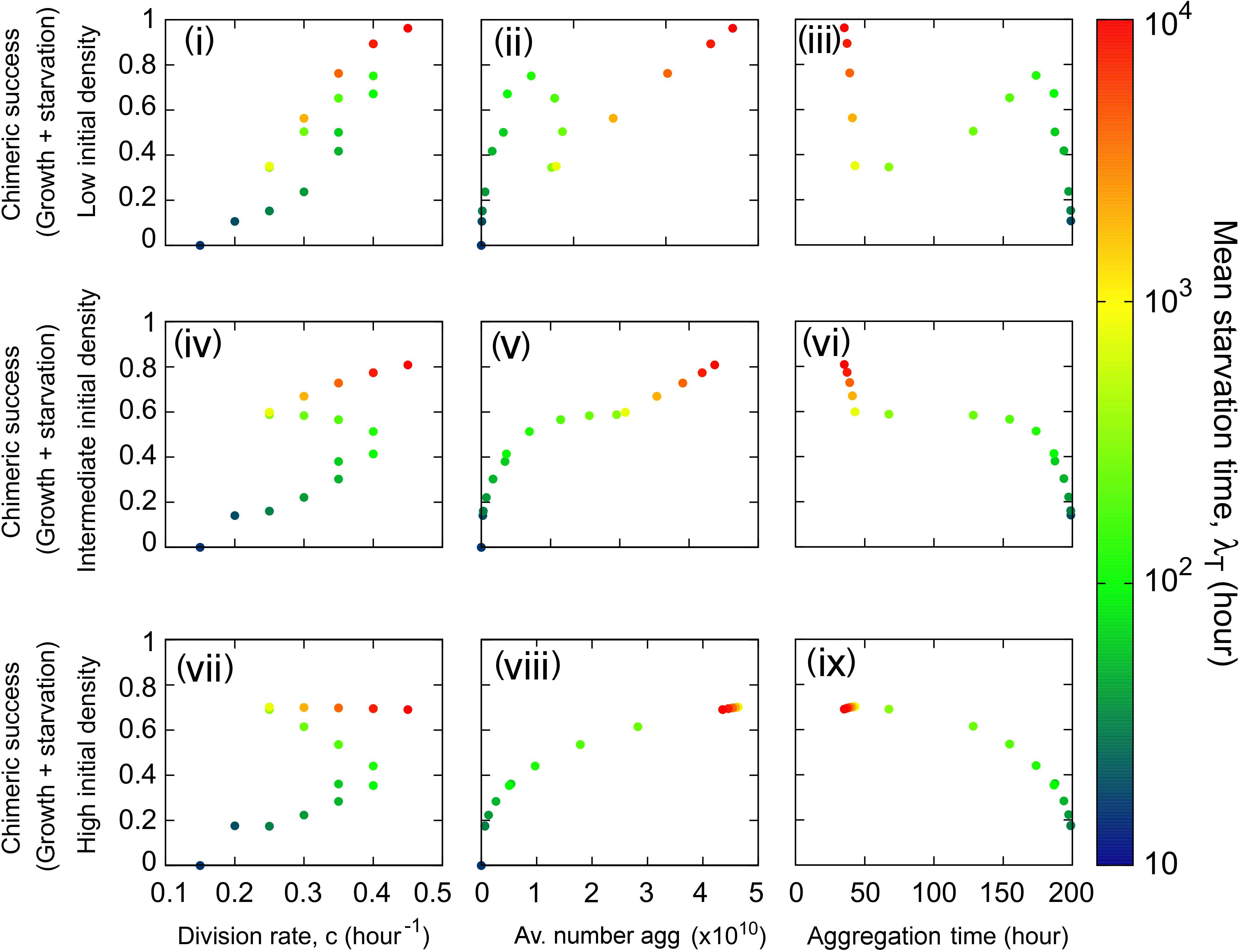


**Fig. D Correlations between chimeric success and non-social traits.** Each one of the 31 winning genotypes obtained in Fig B(ii) is mixed in pairs with the rest of the genotypes and its frequency in the total population of spores at the end of a growth-starvation cycle is counted (200 hours of starvation). The chimeric success is obtained as the mean value of this frequency among the spores averaged over all the possible pair mixes as described by Eq. (5) in the main text. From left to right: average chimeric success versus division rate (A, D, G), average number of aggregators (B, E, H) and aggregation time (C, F, I). From top to bottom: low cell:resource initial density (10^3^ cells and R_0_ = 10^8^), intermediate cell:resource initial density (10^7^ cells and R_0_ = 10^8^) and high cell:resource initial density (10^10^ cells and R_0_ = 10^8^).
